# Loss of Protein Kinase Novel 1 (PKN1) is associated with mild systolic and diastolic contractile dysfunction, increased phospholamban Thr^17^ phosphorylation, and exacerbated ischaemia-reperfusion injury

**DOI:** 10.1093/cvr/cvx206

**Published:** 2017-10-16

**Authors:** Asvi A Francois, Kofo Obasanjo-Blackshire, James E Clark, Andrii Boguslavskyi, Mark R Holt, Peter J Parker, Michael S Marber, Richard J Heads

**Affiliations:** 1 Department of Cardiology, School of Cardiovascular Medicine and Sciences, British Heart Foundation Centre for Research Excellence, Faculty of Life Sciences and Medicine, The Rayne Institute, King’s College London, St Thomas’s Hospital, Lambeth Palace Road, London, UK; 2 Randall Division of Cell and Molecular Biophysics, King’s College London, New Hunt’s House, Guy’s Hospital Campus, London, UK; 3 Division of Cancer Studies, School of Cancer and Pharmaceutical Sciences, Faculty of Life Sciences and Medicine, King’s College London, New Hunt’s House, Guy’s Hospital Campus, London, UK; 4 Protein Phosphorylation Laboratory, Francis Crick Institute, Lincoln’s Inn Fields, London, UK

**Keywords:** Protein kinase Novel 1 (PKN1) • Cardioprotection • Infarction • CamKIIδ • Phospholamban

## Abstract

**Aims:**

PKN1 is a stress-responsive protein kinase acting downstream of small GTP-binding proteins of the Rho/Rac family. The aim was to determine its role in endogenous cardioprotection.

**Methods and results:**

Hearts from PKN1 knockout (KO) or wild type (WT) littermate control mice were perfused in Langendorff mode and subjected to global ischaemia and reperfusion (I/R). Myocardial infarct size was doubled in PKN1 KO hearts compared to WT hearts. PKN1 was basally phosphorylated on the activation loop Thr^778^ PDK1 target site which was unchanged during I/R. However, phosphorylation of p42/p44-MAPK was decreased in KO hearts at baseline and during I/R. In cultured neonatal rat ventricular cardiomyocytes (NRVM) and NRVM transduced with kinase dead (KD) PKN1 K^644^R mutant subjected to simulated ischaemia/reperfusion (sI/R), PhosTag^®^ gel analysis showed net dephosphorylation of PKN1 during sI and early R despite Thr^778^ phosphorylation. siRNA knockdown of PKN1 in NRVM significantly decreased cell survival and increased cell injury by sI/R which was reversed by WT- or KD-PKN1 expression. Confocal immunofluorescence analysis of PKN1 in NRVM showed increased localization to the sarcoplasmic reticulum (SR) during sI. GC-MS/MS and immunoblot analysis of PKN1 immunoprecipitates following sI/R confirmed interaction with CamKIIδ. Co-translocation of PKN1 and CamKIIδ to the SR/membrane fraction during sI correlated with phospholamban (PLB) Thr^17^ phosphorylation. siRNA knockdown of PKN1 in NRVM resulted in increased basal CamKIIδ activation and increased PLB Thr^17^ phosphorylation only during sI. *In vivo* PLB Thr^17^ phosphorylation, Sarco-Endoplasmic Reticulum Ca^2+^ ATPase (SERCA2) expression and Junctophilin-2 (Jph2) expression were also basally increased in PKN1 KO hearts. Furthermore, *in vivo* P-V loop analysis of the beat-to-beat relationship between rate of LV pressure development or relaxation and end diastolic P (EDP) showed mild but significant systolic and diastolic dysfunction with preserved ejection fraction in PKN1 KO hearts.

**Conclusion:**

Loss of PKN1 *in vivo* significantly reduces endogenous cardioprotection and increases myocardial infarct size following I/R injury. Cardioprotection by PKN1 is associated with reduced CamKIIδ-dependent PLB Thr^17^ phosphorylation at the SR and therefore may stabilize the coupling of SR Ca^2+^ handling and contractile function, independent of its kinase activity.

## 1. Introduction

Myocardial ischaemia and reperfusion induce cardiomyocyte injury resulting in cell death-the extent of which is dependent on the length of the ischaemic insult. The myocardium has innate endogenous protective mechanisms which mitigate against injury which are dependent on the activation of kinase signalling and contribute to both basal and inducible cardioprotection.^1–5^

PKN1 is a stress-responsive kinase and a member of the protein kinase novel (PKN) family also known as protein kinase C-related kinases (PRKs).^6^^,^^7^. PKNs currently comprise three isoforms, PKN1, PKN2, and PKN3 (formerly known as PKN or PKN1, PKN2, and PKNβ, respectively) PKN1 and PKN2 are expressed ubiquitously.^7^^,^^8^ The C-terminal kinase domains of PKNs are closely related to those of PKC, having approximately 50% homology with PKCδ and PKCε, but have a quite different regulatory domain, comprising an HR1 (a, b, c) domain, followed by a C2-related domain. Overall these proteins have a domain organization related to that of the yeast PKC-related proteins.^9^ The HR1 domain interacts with the small GTPases Rho (A, B)^10–12^ and Rac^11–13^ when bound to GTP (i.e. in their active state), such that PKNs can act as molecular effectors of these small GTPases. The interaction of Rho with PKN1 induces a conformational change in PKN1 leading to activation loop phosphorylation by 3-Phosphoinositide-Dependent Kinase-1(PDK1) on Thr^774^ in the case of human PKN1 (Thr^778^ in mouse/rat) which is necessary for the catalytic activation of PKN1/2^14^ and critical for the stability of the protein.^15^^,^^16^ Analysis of rat liver PKN1 by mass spectroscopy has also revealed numerous additional sites which are phosphorylated under basal conditions.^17^ Interestingly, many of the phosphorylated serine and threonine residues are in the N-terminal regulatory region and the linker region immediately upstream of the kinase domain.^17^ The functional significance of these sites is not fully understood, but may be important in PKN1 localization.

Hyper- and hypo-osmotic stress induce Rac1- and RhoA-PDK1-dependent PKN1 translocation/activation, respectively,^18–20^ and PKN1 catalytic activity may be required for turnover/exit from membrane localization. However, the role of PKN1 in cardiac ischaemia/reperfusion injury and post-infarction remodelling remains to be explored in detail. Other studies have also supported a role for PKN1 in ischaemia. A PKN1 constitutively active fragment is generated by caspase mediated cleavage of PKN1 under ischaemic conditions *in vivo*^21^^,^^22^ and ischaemic stress promotes PKN1 translocation from the cytosol to the nucleus suggesting that PKN1 may have a role in regulating gene expression.^23^

Recent work has shown that cardiomyocyte-specific transgenic overexpression of constitutively active PKN1 in the heart leads to stable hypertrophy, reduced infarct size, and reduced TUNEL staining following ischaemia/reperfusion, whilst overexpression of dominant negative PKN (K^644^D) has the opposite effect.^24^ This leads to the conclusion that the protective effect of PKN is cell autonomous in cardiomyocytes. The results we show here are that the loss of PKN1 expression by homologous recombination reduces basal p42/p44-MAPK (ERK1/2) phosphorylation without effect on p38-MAPK or p46/p54-SAPK/JNK activation by ischaemia/reperfusion but increases infarct size in support of a cardioprotective function for PKN1. In keeping with this, overexpression of PKN1 in isolated, cultured cardiac myocytes protected against simulated ischaemia/reperfusion injury, whereas knockdown of endogenous PKN1 using siRNA increased the severity of injury. However, in contrast to Ref. 24, loss of kinase activity by introduction of a K^644^R mutation did not alter the protective effect of PKN1 overexpression. The protective effect of PKN1 was associated with an SR localization and association with several ER/SR calcium handling proteins including CamKIIδ during ischaemia. Furthermore, loss of PKN1 resulted in the increased phosphorylation of the CamKIIδ substrate PLB on Thr^17^ during ischaemia. PKN1 loss *in vivo* resulted in mild systolic and diastolic dysfunction at baseline associated with constitutive PLB Thr^17^ phosphorylation as well as increased levels of the SR Ca^2+^ pump SERCA2. These results suggests a role for PKN1 in the maintenance of SR Ca^2+^ regulatory processes in normal hearts which limits the development of ischaemia/reperfusion injury, but that these effects are independent of its kinase activity.

## 2. Methods

All animal experiments were performed in accordance with European Commission and UK Home Office guidelines and were approved by the local University animal ethics review panel.

### 2.1 PKN1 knockout mouse

The PKN1 global knockout (KO) mouse line was generated by homologous recombination with insertion of a neomycin cassette into exon 2 of the PKN1 gene. PKN1 knockout mice were generated at the Cancer Research UK, London Research Institute (now the Francis Crick Institute), Lincoln‘s Inn Fields, London as previously described.^25^

### 2.2 Antibodies

Monoclonal primary antibody against PKN1 was obtained from BD-Transduction Labs, UK. Polyclonal antibodies for phospho-PRK1 (Thr^774^)/PRK2 (Thr^816^), DYKDDDK (FLAG^®^), Nogo-A, phospho-Erk (Thr^202^/Tyr^204^), Erk 1/2, phospho-JNK (Thr^183^/Tyr^185^), JNK, phospho-p38 (Thr^180^/Tyr^182^), p38, GAPDH were from Cell Signalling Technologies, UK. Polyclonal antibody against p-CamKIIδ (Thr^287^) was from Life Technologies, UK. Polyclonal antibodies against CamKIIδ and Jph2 were from Abcam, UK. Polyclonal antibodies against SERCA2a, phospho-phospholamban (Thr^17^), and total phospholamban were from Badrilla, UK. Monoclonal antibody against Na^+^/K^+^ATPase alpha (NKAα) was from ThermoFisher, UK. Monoclonal sarcomeric α-actinin antibody was obtained from Sigma-Aldrich, UK. Cy3- and Cy5-congugate antibodies were from Jackson Laboratories.

### 2.3 Plasmid constructs

A construct encoding human PKN1 cDNA with a C-terminal FLAG^®^ tag was generated using a GFP-PKN1 template plasmid.^18^ PKN1 was amplified using primer 1: 5’-GCGCAAGCTTGCATGGCCAGCGACGCCGTGC-3’ and primer 2: 5’-TCAATGTACGGTACCTCTACTTATCGTCGTCATCCTTGTAATCGCAGCC-3’. Primer 1 incorporates a *Hind*III restriction site whereas primer 2 encodes a FLAG^®^ epitope and a *Kpn*1 restriction site. The product was digested with *Hind*III and *Kpn*1 and subsequently cloned into pCMVScript (Promega). The resulting plasmid contained the full length PKN1 sequence fused to a C-terminal FLAG^®^ tag (PKN1_FLAG). The PKN1_FLAG sequence was transferred to a small shuttle plasmid (pDONR221) using the Gateway^®^ cloning technology (Life Technologies) according to the manufacturer‘s instructions. Briefly, PKN1_FLAG, the CMV promoter and the translation termination sequence from pCMVScript were amplified using primer 3: 5’-GGG GAC AAGTTT GTA CAA AAA AGC AGG CTA TGC ATT AGT TAT TAA TAG TAA TCA ATT ACG GGG TC-3’ and primer 4: 5’-GGG GAC CAC TTT GTA CAA GAA AGC TGG GTC GCG AAT TTT AAC AAA ATA TTA ACG CTT ACA ATT TAC-3’. Primers 3 and 4 encode 5’- and 3’- att B sites for subsequent recombination. The product from this step was combined with pDONR221 in the presence of BP Clonase II (Life technologies) to generate recombinant pDONR221 encoding PKN1_FLAG and components required for its transcription and translation from this plasmid (pDONR-PKN1_FLAG). The kinase dead mutant (KDPKN1_FLAG) was generated by introducing a single mutation (K^644^R) by site directed mutagenizes using primer 5: 5’-GGG AGC TGT TCG CCA TCA GGG CTC TGA AG-3’ and primer 6: 5’- CTT CAG AGC CCT GAT GGC GAA CAG CTC CC-3’ using pDONR-PKN1_FLAG as a template. As a negative control, an empty vector encoding FLAG^®^ but not PKN1 was generated in pDONR221 (mock) using a similar strategy as described above. Finally, to generate adenoviral pacmid constructs, pDONR plasmid (PKN1_FLAG, KD-PKN1_FLAG, mock) was recombined with pAd-DESTPL using LR Clonase II (Life Technologies) according to the manufacturer‘s instructions. Products from each step were verified by sequence analysis.

### 2.4 Isolation and culture of neonatal rat ventricular myocytes

Neonatal rats were euthanized by cervical dislocation according to schedule 1 of the UK Home Office guidelines. Ventricular cardiomyocytes (NRVMs) were isolated from 1–2 day old Sprague-Dawley rat hearts by collagenase/pancreatin digestion as described previously.^3^ Cells were plated at a density of approximately 1 x 10^6^ cells/well on gelatin coated 6-well plates (NUNC) in medium containing 4: 1 DMEM: M199 supplemented with 5% foetal calf serum; 10% horse serum and 1% penicillin/streptomycin (GIBCO) for 24 h and then transferred to maintenance medium (MM) (serum-free DMEM: M199 plus antibiotics).

### 2.5 RNA interference studies

For PKN1 knockdown studies, siRNA or adenovirus harbouring shRNA were used. For siRNA experiments commercially available, pre-designed siRNA against rat Pkn1 (Silencer^®^ Select 4390771) and an siRNA oligomer predicted not to target any known mammalian gene (Silencer^®^ Select Negative Control #1) were obtained from Life Technologies. For transfection, 15 nM siRNA was complexed with Lipofectamine RNAiMAX (Life Technologies) according to the manufacturer‘s instructions in Opti-MEM (Life Technologies). Nuclear complexes were applied on cells in 1.0 mL of transfection solution (116 mM NaCl, 1 mM NaH_2_P0_4_, 0.8 mM MgSO_4_, 5.5 mM glucose, 32.1 mM NaHCO_3_, 1.8 mM CaCl_2_, pH7.2) supplemented with M199 (4:1), 4% (v/v) horse serum) for 5 h at 37 °C. Experiments were carried out 48 h post transfection.

For shRNA experiments shRNA harbouring adenovirus were generated using the BlockiT kit (Life Technologies). Two oligonucleotides specifically targeting rat Pkn1 mRNA were designed *in silico* using the BlockiT RNAi designer algorithm (Life Technologies): shRNA1 (GGATAGTAAGACCAAGATTGA), shRNA2 (GGAAGACTTCTTGGACAATGA). As a negative control, two oligomers encoding a scramble sequence predicted not to target any known mammalian gene were designed as described above: NC1 (GGAATGGACAAGCAATAAGTT), NC2 (GCTATACTTCTACGACTATGC). Oligonucleotides were cloned into pADDESTPL for subsequent adenoviral generation according to the manufacturer‘s instruction.

### 2.6 Adenoviral gene transfer

Adenoviral constructs in pADDESTPL were generated as described above. Adenovirus were generated according to the BlockiT Adenoviral kit (Life Technologies). Briefly, linearized pacmid DNA was transfected in HEK293 cells using Lipofectamine 2000 (Life Technologies) and resulting adenovirus were isolated using repeated freeze-thaw cycles. Adenoviral titre was determined using the AdEasy Viral Titre kit (Agilent Technologies). NRVMs were infected 48 h after isolation in maintenance media. Media was replaced 24 h after infection.

### 2.7 Simulated ischaemia (sI)

Sublethal SI was induced by treating NRVMs with a modified Krebs buffer containing (in mM): 137 NaCl; 12 KCl; 0.49 MgCl_2_; 1.8 CaCl_2_; 4 HEPES supplemented with 10 mM 2-deoxyglucose; 20 mM Na lactate; and 1 mM oxygen scavenger (sodium dithionite) pH 6.8. to simulate the extracellular milieu of myocardial ischaemia as described previously.^3^ Briefly, cells in 6-well tissue culture plates were exposed to 1.0 mL per well SI buffer at 37.0 °C. Simulated reperfusion was achieved by removing the SI buffer and replacing with maintenance media (MM) for the indicated time.

### 2.8 Gel electrophoresis and Western blotting

**Figure 1:**
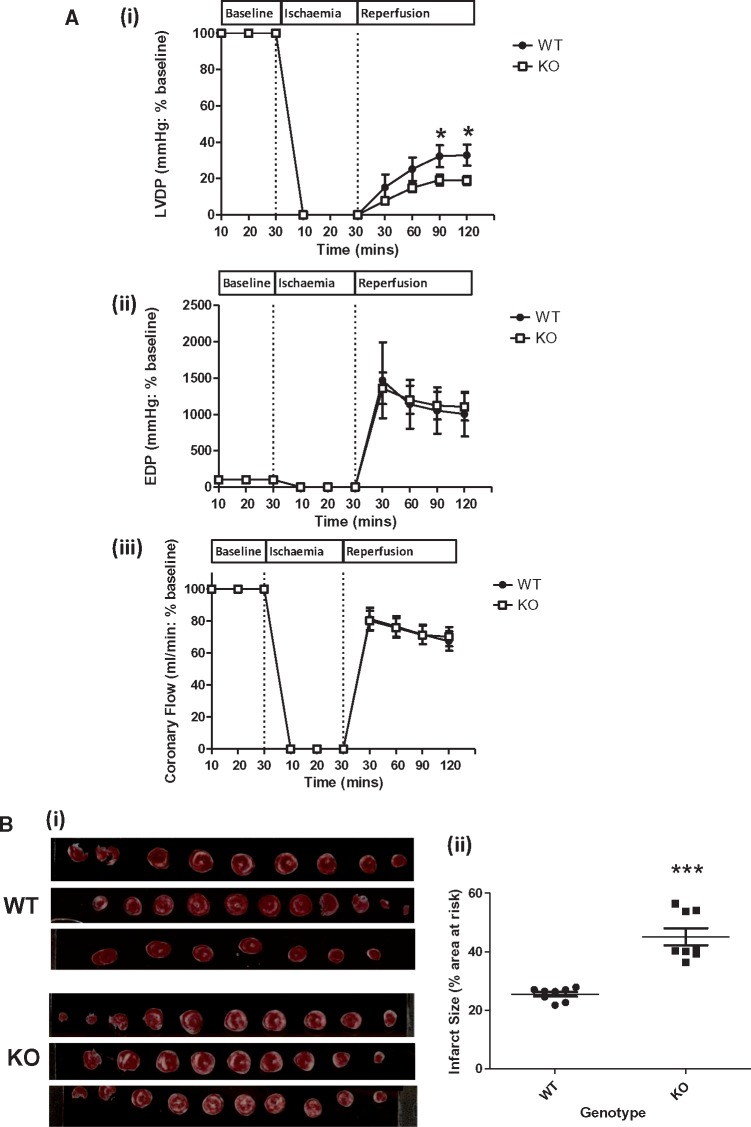
*Ischaemia-Reperfusion Injury (Infarct size) is Increased in PKN1 Knockout hearts.* (*PanelA*) Haemodynamics in WT and PKN1 KO Hearts. Hearts were stabilized during 30 min aerobic perfusion followed by 30 min global ischaemia followed by 120 min reperfusion. (i–iii): Haemodynamic parameters of isolated buffer-perfused hearts from matched littermate wild type (WT) and PKN1 knockout (KO). Values are presented as mean ± SEM (*n* = 8) for left ventricular developed pressure (LVDP), end diastolic pressure (EDP), and coronary flow (CF) recorded following 30 min stabilization, 30 min global ischaemia, and 120 min reperfusion. LVDP was significantly reduced in the PKN1 KO hearts. Statistical analysis was by Two-Way ANOVA with a Bonferroni post-hoc test, where significance is expressed as *P ≤ 0.05 vs. WT. (*PanelB*) (i): Representative images showing triphenyl tetrazolium chloride (TTC) staining of individual WT and PKN1 KO heart slices. Viable tissue is stained red and non-viable (necrotic) tissue appears white. (ii): Infarct volume as a percentage of area at risk (total heart volume) in age matched male littermate WT and PKN1 KO hearts subjected to 30 min global ischaemia and 2 h reperfusion. Results are expressed as mean ± SEM (*n* = 8). Statistical analysis was by One Way ANOVA with a Newman-Keuls post-hoc test, where ***P ≤ 0.001.

**Figure 2:**
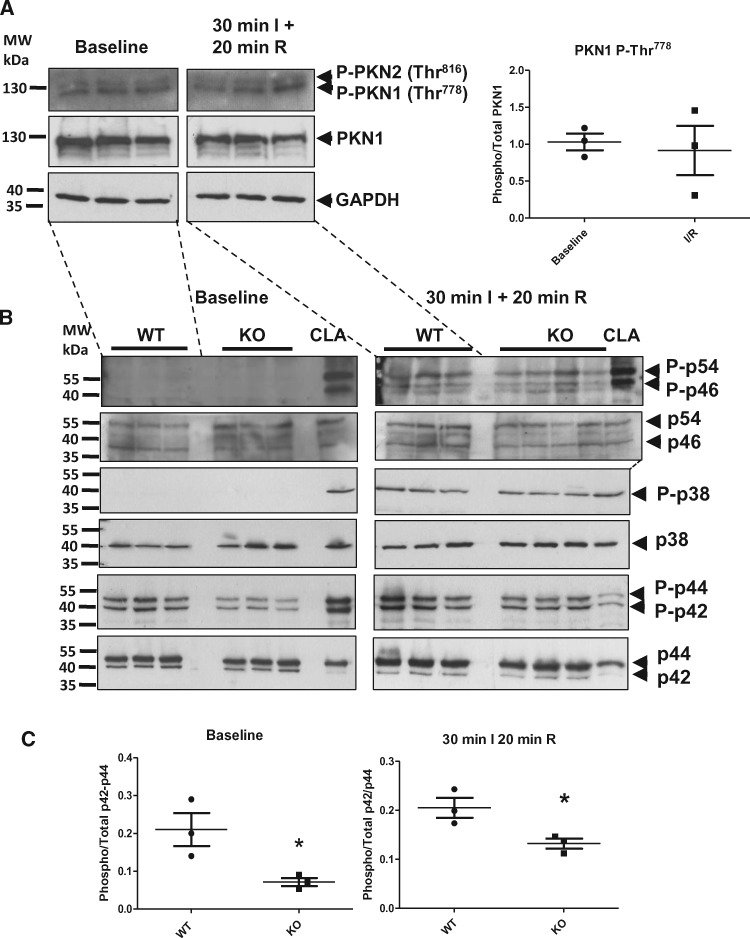
*PKN1 Thr^778^ Phosphorylation is Unchanged During Ischaemia-reperfusion, but p42/p44-MAPK Phosphorylation is Reduced in PKN1 Knockout Hearts.* (*PanelA*) Wild type c57BL/6 mouse hearts were perfused in Langendorff mode and were subjected to 30 min global ischaemia and 20 min reperfusion. Hearts were harvested after 30 min of baseline perfusion or 30 min SI + 20 min reperfusion by freeze-clamping followed by homogenization and processing for SDS-PAGE and Western immunoblotting as described in the methods section. PKN1 activation loop phosphorylation (Thr^778^) was assessed by probing blots with anti-phospho-PKN1/2 (Thr^774^/Thr^816^) or anti-total PKN1 antibody. Total GAPDH was used as an additional control for loading. Phospho-PKN was quantitated by densitometry and normalized to total PKN and compared using a two-tailed unpaired *t*-test (*n* = 3 individual hearts). (*PanelB*) Matched littermate wild type (WT) and PKN1 knockout (KO) mouse hearts were perfused in Langendorff mode and subjected to 30 min global ischaemia followed by 20 min reperfusion. Hearts were harvested and processed as above and MAPK and SAPK activation was assessed using antibodies recognizing dually phosphorylated p46/p54-SAPK (JNK), p38-MAPK, and p42/p44-MAPK (ERK1/2) (upper panels) or the corresponding total protein (lower panels). (*PanelC*) Phospho-p42/p44-MAPKs (P-ERK1/2) were quantitated by densitometry and normalized to total p42/p44-MAPKs (T-ERK1/2) and compared using a two-tailed unpaired *t*-test (*n* = 3 individual hearts). Each sample represents a different lysate prepared from individual hearts exposed independently to ischaemia/reperfusion. Lysates from NRVMs treated with 50 nM calyculin A (CLA) were used as a positive control for phospho-p42/44-MAPK, phospho-p46/p54-SAPK (JNK), and phospho-p38-MAPK.

**Figure 3:**
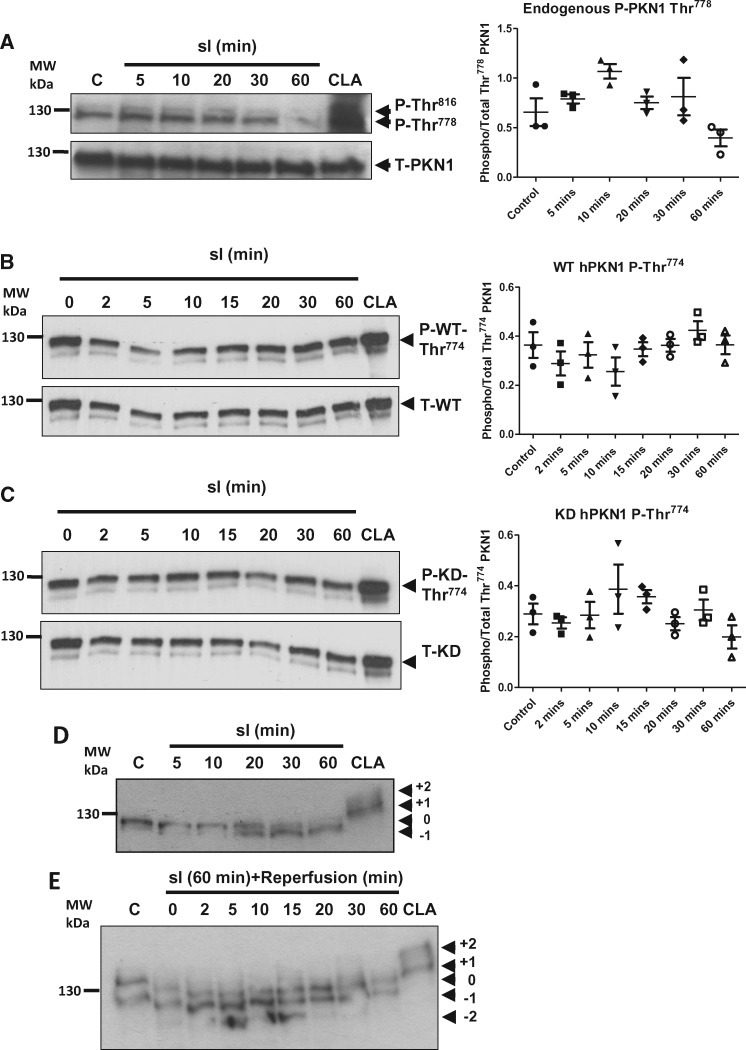
*Thr^774/778^ Phosphorylation of PKN1 is Unchanged but Net Dephosphorylation Occurs on Other Sites During Simulated Ischaemia/Reperfusion (sI/R) in Isolated Cardiomyocytes.* (*Panel A*) NRVMs were treated with SI for up to 1 h and harvested at the indicated times in 2x sample buffer. Phosphorylation of endogenous PKN1 was analysed by Western immunoblotting and probed with antibodies against phospho-PKN1 (Thr^778^) or total PKN1. (*Panel B*) NRVMs were transfected with wild type- (WT) hPKN1-FLAG, treated with SI for up to 60 min and harvested at the indicated times. Samples were analysed by Western immunolotting to determine hPKN1 Thr^774^ phosphorylation and compared to levels of total PKN1 (T-hPKN1). Phospho blots were stripped and re-probed with anti-total PKN1. (*Panel C*) NRVMs were transfected with kinase dead (KD: K^644^R)-hPKN1-FLAG, treated with SI for up to 60 min and harvested at the indicated times. Samples were analysed by Western immunolotting to determine hPKN1 Thr^774^ phosphorylation and compared to levels of total PKN1 (T-hPKN1). Phospho blots were stripped and re-probed with anti-total PKN1. (*Panel D*) NRVMs were treated with SI for up to 1 h and harvested as indicated in 2x sample buffer and analysed by PhosTag^®^ SDS-PAGE followed by western immunoblotting as described in the materials and methods. Immunoblots were probed with monoclonal antibody against total PKN1. (*Panel E*) NRVMs were treated with SI for 1 h, ‘reperfused’ and harvested at the indicated times of ‘reperfusion’ and analysed by PhosTag^®^ SDS-PAGE followed by Western immunoblotting. Immunoblots were probed with monoclonal antibody against total PKN1 as for panel A. For all panels representative images are shown for one of three independent experiments. In each case, NRVMs were treated with 50 nM CLA as a positive control for maximal PKN1 phosphorylation.

Protein extracts were separated by SDS-PAGE using a mini-protean II apparatus (Biorad, UK) essentially as previously described,^3^ except that for resolution of phospho- and total-PKN1 and PKN2, 6% gels were used (*Figures 2A, 3A, 3B* and *3C*). For experiments analysing immunoprecipitations and heart extracts for phospho- and total-phospholamban (PLB), pre-cast 4-20% gradient gels (ThermoScientific, UK) were used (*Figures 7, 8, 9*). For the analysis of heart extracts for phosphor- and total-MAPK/SAPK, 12.5% gels were used (*Figure 1B*). Western blotting was performed as previously described^3^ following electrophoretic transfer onto PVDF membranes (Hybond-P^®^, GE Healthcare, UK) using a mini-Protean II Transblot apparatus (Biorad, UK). Membranes were blocked in 2% (w/v) powdered milk (Marvel^®^) in PBS; 0, 05% Tween 20 (PBST), incubated with primary antibodies for 3 h at RT at 1:1000 dilution in PBST; 0.1% milk (PBSTM), washed 3 x 5 min in PBSTM, incubated with the appropriate HRP-conjugated secondary antibodies (DAKO, UK) for 1 h at RT at 1:2500 dilution in PBSTM, washed 3 x 5 min in PBSTM and developed with ECL enhanced chemiluminescence reagent (GE Healthcare, UK). Exceptions were anti-total PLB and anti-SERCA2a which were used at 1:4000 and 1:5000 dilution, respectively. Where indicated (Figure legends) phosphor-blots were stripped with 0.5 M NaOH for 30 min at RT followed by several washes in ddH_2_O prior to re-probing with pan (total) kinase antibody. For PhosTag™ gel analysis of PKN1 phosphorylation, samples were run on 8% gels supplemented with PhosTag™ acrylamide (Wako Chemicals, Germany) and MnCl_2_ to a [final] 40 μM. Gels were electrophoresed at a constant 90 V. 

### 2.9 Determination of NRVM injury

Cell injury following SI and reperfusion was determined by creatine phosphokinase (CPK) efflux (membrane damage) and methyl thiazoyltetrazolium (MTT) metabolism (cell viability) as described previously.^3^ NRVMs were exposed to 1 h of SI then reperfused in 1.0 mL of MM. 2 h after reperfusion, the media was collected and assessed for CPK activity using a commercially available kit (Abnova, Taiwan) according to the manufacturer‘s instructions. Fresh MM was applied to the NRVMs and allowed to reperfuse for a further 18 h. Cell viability was measured using an MTT assay as previously described.^3^ Cells were washed in warm PBS and incubated with 0.5 mg/mL MTT in PBS for 30 min at 37 °C. The reaction was stopped by the addition of an equal volume of solubilization solution [0.1 N HCl, 10% (v/v) Triton X-100 in isopropanol], and the absorbance of the blue formazan derivative read at 570 nm.

### 2.10 Immunofluorescence experiments

Cells were fixed in 4% paraformaldehyde, permeabilized with 0.2% (v/v) Triton X-100 and blocked with 2% (w/v) BSA in PBS. Cells were incubated with primary antibodies overnight at 4 °C. Cy3- or Cy5-coupled secondary antibodies were applied for 2 h at room temperature. Cells were mounted with VectaShield containing DAPI and visualized using Leica SP5 confocal microscope. For quantitation of PKN1 SR localization, 16 bit confocal images were imported in to Wolfram Mathematica v11.0 (Champaign, IL) and were background subtracted using a 51 pixel radius Gaussian kernel. The images were then bandpass filtered at a frequency corresponding to the spacing of the SR and with a quality factor of 1 using the inbuilt ‘BandpassFilter’ command. This filtered image was binarized to create a mask for SR resident protein and an inverted version for non-SR protein. These masks were then multiplied by the background subtracted images. The total intensity was then calculated from these images to give the total amount of protein (in arbitrary units) in both pools. The ratio of SR localized protein to total protein was then calculated. This analysis was performed blinded to genotype.

### 2.11 Immuno-precipitation

PKN1-FLAG was immunoprecipitated from NRVMs using ANTI-FLAG^®^ M2 Affinity gel (Sigma-Aldrich) according to the manufacturer‘s instruction. Briefly, NRVMs were lysed in ice-cold Lysis Buffer [50 mM Tris HCl, pH7.4; 150 mM NaCl; 1 mM EDTA; 1% (v/v) TRITON X-100; cOmplete protease inhibitor cocktail tablet (Roche)]. Lysates were centrifuged for 1 min at 1000 x g at 4.0 °C to remove cell debris and then incubated with the affinity gel for 2 h at 4.0 °C with gentle agitation. After washing the beads in cold TBS, PKN1_FLAG was eluted by boiling in 2x sample buffer for 3 min. 20% (v/v) β-mercaptoethanol was added before SDS-PAGE analysis.

### 2.12 Langendorff perfused murine heart preparation

Age-matched 10–12 week old male PKN^+/+^ (WT) and PKN^-/-^ (KO) littermate mice were anesthetized with pentobarbital sodium in combination with heparin (200 mg/kg and 200 IU/kg, respectively, ip). Hearts were rapidly excised and placed in cold (4.0 °C) K-H buffer (118.5 mM NaCl; 25.0 mM NaHCO_3_; 1.18 mM KH_2_P0_4_; 1.19 mM MgSO_4_.7H_2_0; 11 mM glucose; 1.04 mM CaCl_2_, pH 7.4), and the aorta was cannulated with a ‘blunted’ 21-gauge needle. Hearts were then perfused with oxygenated (95% 0_2_–5% CO_2_) K-H buffer at 37.0 °C. Perfusion was in the non-recirculating Langendorff mode at a constant pressure equivalent to 80 mmHg, and were paced at 600 beats per min (bpm). Left ventricular developed pressure (LVDP) measurements were performed with a fluid-filled balloon inflated to give an end diastolic pressure of ∼4–9 mmHg. Cardiac performance was further analysed by determination of pressure-volume relationships (PV loop analysis) as previously described in detail.^26^ For this study mice of different genotypes were randomized into the baseline or infarction groups.

### 2.13 Infarct size measurement

At the end of the protocol, hearts were perfused with 1% triphenyl tetrazolium chloride (TTC) in warm K-H buffer (Sigma, UK) for 3 min and immediately fixed in 4.0% formaldehyde overnight at 4.0 °C. Fixed hearts were rinsed in cold PBS, set in 4.0% agarose and sectioned with a Vibratome™ 1000 plus (Products International Inc, USA) at thickness of 0.75 μm. Sections were scanned at 1200 dpi and the infarct area delineated with planimetry using Sigma Scan Pro software and surface area transformed to volume by multiplication with tissue depth. The infarct size was expressed as the percentage of area at risk, defined as the sum of total ventricular area minus cavities. Analysis of infarct size by planimetry was performed blinded to genotype.

### 2.14 Cell fractionation

*NRVMs* were harvested in cold Buffer A [10 mM HEPES, pH 7.4, 0.154 M KCl, 1 mM EDTA, 20% (v/v) glycerol] and lysed by three 12 s bursts of sonication on ice. Cell lysates were centrifuged at 1000 *g* for 10 min at 4 °C. The pellet (P1) was washed in Buffer A and was resuspended in 2x sample buffer. The fraction was denoted as the ‘insoluble fraction’. The supernatant (S1) was centrifuged at 100 000 *g* for 1 h at 4 °C. The supernatant from this step (S2) was denoted ‘Cytosolic fraction’. The pellet from the fast spin (P2) was washed three times in Buffer A and resuspended in the same buffer using a glass-glass homogenizer placed on ice. This was denoted the ‘membrane fraction’. The cytosolic and membrane fractions were prepared in 2x SDS-PAGE sample buffer. All samples were stored at –20 °C until SDS PAGE analysis.

### 2.15 Sample preparation from langendorff perfused hearts

At the end of the protocol, perfused hearts in Langendorff mode were harvested and immediately freeze clamped in liquid nitrogen. Hearts were stored at –80 °C until analysis. Hearts were homogenized in homogenization buffer [50 mM Tris HCl (pH7.4); 1 mM EGTA; 1 mM EDTA; 1% (v/v) TRITON X-100; 0.1% (v/v) β-mercaptoethanol; 50 mM NaF; complete protease inhibitor cocktail tablet (Roche)] to a concentration of 100 mg/mL using a hand held homogenizer for 3 min on ice. An equal volume of 2x loading buffer with 20% (v/v) β-mercaptoethanol was added was and boiled for 5 min at 95.0 °C. Samples were stored at –20.0 °C until further analysis.

### 2.16 LC-MS/MS

Gel bands were excised and pooled prior to digestion, extraction and analysis by mass spectrometry. LC-MS/MS was performed by Mr. Steven Lynham, King‘s College London Centre of Excellence for Mass Spectrometry (CEMS), Institute of Psychiatry, Denmark Hill.

#### 2.16.1 Enzymatic digestion

In-gel reduction, alkylation, and digestion with trypsin were performed prior to subsequent analysis by mass spectrometry. Cysteine residues were reduced with dithiothreitol and derivatized by treatment with iodoacetamide to form stable carbamidomethyl derivatives. Trypsin digestion was carried out overnight at room temperature after initial incubation at 37 °C for 2 h.

#### 2.16.2 LC-MS/MS

Peptides were extracted from the gel pieces by a series of acetonitrile and aqueous washes. The extract was pooled with the initial supernatant and lyophilized. Each sample was then resuspended in 10 μL of 50 mM ammonium bicarbonate and analysed by LC/MS/MS. Chromatographic separations were performed using an EASY NanoLC system (ThermoFisherScientific, UK). Peptides were resolved by reversed phase chromatography on a 75 μm C18 column using a three step linear gradient of acetonitrile in 0.1% formic acid. The gradient was delivered to elute the peptides at a flow rate of 300 nL/min over 180 min. The eluate was ionized by electrospray ionization using an Orbitrap Velos Pro (ThermoFisherScientific, UK) operating under Xcalibur v2.2. The instrument was programmed to acquire in automated data-dependent switching mode, selecting precursor ions based on their intensity for sequencing by collision-induced fragmentation using a Top20 CID method. The MS/MS analyses were conducted using collision energy profiles that were chosen based on the mass-to-charge ratio (*m/z*) and the charge state of the peptide.

### 2.17 Statistical analysis

Statistical analysis was by Two-Way ANOVA with a Bonferroni post-hoc test; One Way ANOVA with a Newman-Keuls post-hoc test or unpaired *t*-test (GraphPad Prism 5^®^) where significance is expressed as *P ≤ 0.05, **P ≤ 0.01, and ***P ≤ 0.001 vs. control as appropriate to the experiment. For confocal analysis of PKN1 co-localization to the SR a nested ANOVA was performed using the RealStatistics™ add-in for MS Excel.

## 3. Results

### 3.1 Characterization of PKN1 null mice

PKN1^-/-^ mice show no expression of PKN1 and no compensatory changes or PKN2 or PKN3 expression (see Supplementary material online, *Figure S1* and Ref. 25). First, we analysed cardiac haemodynamic and functional parameters in WT and PKN1 KO hearts perfused in Langendorff mode, both at baseline (see Supplementary material online, *Figure S2A–C*) and following ischaemia and reperfusion (I/R) [*Figure 1A* (*i*–*iii*)]. There were no differences in baseline haemodynamic parameters between WT and PKN1 KO hearts. Following 30 min global ischaemia and 120 min reperfusion, functional recovery (LVDP) was 33.6 ± 6.1% (*n* = 8) in WT hearts, whereas LVDP recovered significantly less, 17.7 ± 3.9% (*n* = 8) (*P* ≤ 0.05) in PKN1 KO hearts [*Figure 1A* (*i*)]. There no significant difference in end diastolic pressure (EDP) [*Figure 1A* (*ii*)] or coronary flow [*Figure 1A* (*iii*)]. between genotypes. These results reflect increased injury and decreased functional recovery in PKN1 KO hearts following I/R. Comparison of the extent of ischaemic injury (necrosis) was determined and the infarct size (IS) expressed as a percentage of the area at risk (AAR) following triphenyl tetrazolium chloride (TTC) staining. IS in the PKN1 KO hearts was 45.1 ± 2.9% compared to 25.5 ± 0.8% in WT hearts (*P* ≤ 0.0001) [*Figure 1B* (*ii*)]. Together, these results demonstrate that PKN1 KO hearts were significantly more susceptible to I/R injury compared to WT hearts.

We next determined changes in PKN1 phosphorylation on Thr^778^, the activation loop PDK1-dependent site, following ischaemia and reperfusion in hearts from c57Bl/6 mice perfused in Langendorff mode. Hearts were analysed at 20 min of reperfusion following a 30 min ischaemic episode since peak activation loop phosphorylation of canonical MAPKs/SAPKs (p42/p44-MAPK, p46/p54-SAPK/JNK, p38-MAPK) were shown at this time point (see Supplementary material online, *Figure S2*). PKN1 Thr^778^ phosphorylation was relatively high at baseline and not different during I/R at this time point (*Figure 2A*). We next compared the activation loop phosphorylation of the canonical MAPKs/SAPKs at this time point in PKN1 knockout (KO) compared to wild type (WT) littermate control hearts. In accordance with previous studies, I/R induced an increase in p46/p54-SAPK (JNK) and p38-MAPK phosphorylation compared to baseline. However, there were no differences between genotype in p38-MAPK or p46/p54-JNK phosphorylation following I/R. In contrast, p42/p44-MAPK (ERK1/2) Thr^202^/Tyr^204^ phosphorylation was significantly reduced at baseline and also following I/R (*Figure 2B* and *C*).

### 3.2 Molecular changes in PKN1 in isolated ventricular cardiomyocytes

Having established that loss of PKN1 increased susceptibility to I/R injury *in vivo*, we next aimed to characterize the molecular changes in PKN1 using an established model of simulated ischaemia/reperfusion (sI/R) in isolated primary neonatal rat ventricular cardiomyocytes (NRVM).^3^ Canonical MAPK/SAPK phosphorylation patterns in NRVM in response to sI/R were consistent with those see during global ischaemia and reperfusion in isolated hearts (see Supplementary material online, *Figure S3*). Firstly we examined whether there are dynamic changes in endogenous PKN1 Thr^778^ phosphorylation during sI/R, given that basal Thr^778^ phosphorylation was unchanged following 30 min I/20 min R in isolated hearts (*Figure 2A*). *Figure 3A* shows that the basal activation state of endogenous PKN1 as determined by Thr^778^ phosphorylation was relatively high in NRVM under these conditions. Thr^778^ phosphorylation was unchanged during sI until at least 30 min. However, following extended sI to 60 min, Thr^778^ phosphorylation was subsequently lost. This is consistent with a general loss of global kinase activity due to run-down of cellular ATP levels with extended ischaemia times. Following loss of Thr^778^ phosphorylation after 60 min sI, Thr^778^ phosphorylation recovered during ‘reperfusion’ (results not shown). The antibody used to detect endogenous Thr^778^ phosphorylation also recognizes the equivalent activation loop phosphorylation site, Thr^816^, in PKN2. PKN2 Thr^816^ phosphorylation showed parallel changes to PKN1 Thr^778^.

To determine whether PKN1 stability or Thr^774/778^ phosphorylation was dependent on PKN1 kinase activity itself, we introduced an active site mutation (K^644^R) into the human PKN1 cDNA to render it kinase dead (KD-hPKN1-FLAG). NRVM were transduced with adenoviruses expressing WT- or KD-hPKN1 and subjected to sI. As shown in *Figure 3B* Thr^774^ phosphorylation of the transfected WT-hPKN1 was unaffected by sI up to 60 min. Furthermore, Thr^774^ phosphorylation was also unaffected by the K^644^R mutation (*Figure 3C*). Changes in phosphorylation of the PKN1 activation loop Thr^774/778^ reflect the upstream PDK1 activity. However, it is also likely that changes in other phosphorylation sites on PKN1 may regulate PKN1 kinase activity and/or localization during I/R. The role of other possible regulatory phosphorylation sites in PKN1 have not been characterized to date. To gain an insight into global changes in PKN1 phosphorylation we analysed samples from sI/R timecourses using PhosTag^®^ gels in which protein mobility is retarded by adduction of the PhosTag^®^ reagent to phosphorylated residues on the target protein, as detected using a pan-antibody on Western blots. The reduced mobility of PKN1 in cells treated with the phosphatase inhibitor calyculin A (positive control) showed that there are multiple phosphorylation sites on PKN1 which have the potential to be dynamically regulated by cellular kinase/phosphatase activity (*Figure 3D/E*). Under control conditions PKN1 appeared as a doublet. During sI the accumulation of the lower, faster migrating band (designated -1) occurred between 20–60 min sI (*Figure 3D*) indicative of net dephosphorylation of PKN1 during sI. Interestingly, rather than recovery of the basal phosphorylation state during early reperfusion, we observed further accumulation of the –1 species and the appearance of an additional faster migrating band (designated -2) between 5–15 min of recovery, indicative of further dephosphorylation (*Figure 3E*). The basal state of PKN1 phosphorylation was then re-established at later recovery times (20–60 min). These results demonstrate that whereas Thr^778^ phosphorylation is unchanged during sI and subsequent reperfusion, phosphorylation of other sites is very dynamic during I/R.

### 3.3 Role of PKN1 in cellular injury

**Figure 4:**
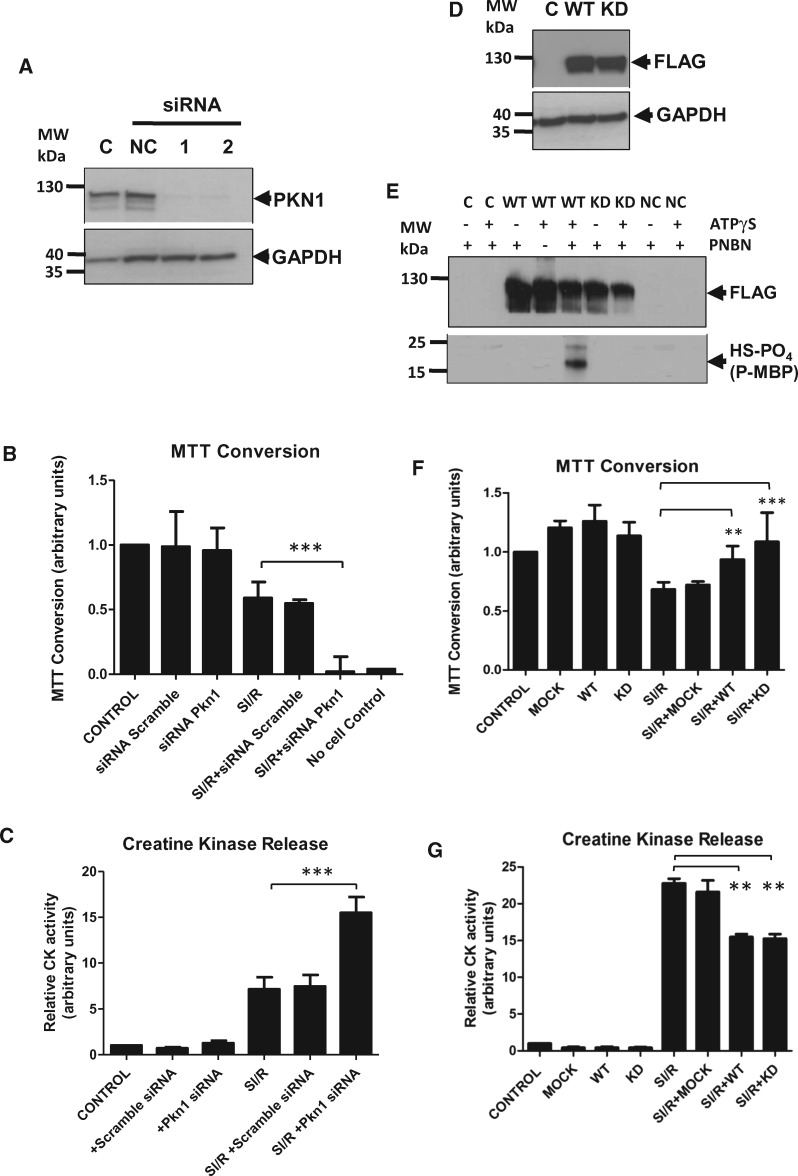
*PKN1 is Protective Against Simulated Ischaemia/Reperfusion in Cardiomyocytes.* NRVMs in which PKN1 was either silenced by siRNA treatment (**Panels*A–C*) or overexpressed by adenoviral infection (WT-hPKN1-FLAG or KD-hPKN1-FLAG) (**Panels*D–G*) were exposed to 1 h SI followed by 18 h of ‘reperfusion’. (*PanelA*) The efficacy of siRNA knockdown of PKN1 as analysed by western blot. (*PanelD*): The efficacy of overexpression of FLAG-tagged wild type (WT) and kinase dead (KD) PKN1 as assessed by western blot using an anti-FLAG antibody. (*PanelE*) Cells were transfected with WT- or KD-hPKN1-FLAG and treated with 0.5 M sorbitol for 30 min to activate PKN1. Cell lysates were prepared and IPd with anti-FLAG. Cell lysates were prepared and *in vitro* kinase assays performed using ATPγS showing kinase activity of WT-hPKN-FLAG but not KD-hPKN1-FLAG towards a myelin basic protein (MBP) substrate. An anti-phosphothioate antibody was used for detection of MBP phosphorylation following Western blotting. (*PanelsB* and *F*) Cell viability was assessed by the conversion of MTT as described in the materials and methods section Results represent mean +/- SEM from four individual experiments in which each group was tested in triplicate. Values from the control group were set as 1 and test groups were normalized to this. Statistical significance of differences compared with SI/R: *P ≤ 0.05, **P ≤ 0.01, ***P ≤ 0.001. Cell injury was assessed by the activity of CPK as described in the materials and methods section (*PanelsC* and *G*). Results represent mean +/- SEM from four individual experiments in which each group was tested in triplicate. Values from the control group were set as 1 and test groups were normalized to this. Statistical significance of differences compared with SI/R: *P ≤ 0.05, **P ≤ 0.01, ***P ≤ 0.001 using One-Way ANOVA with a Newman-Keuls post-hoc test.

In order to recapitulate the *in vivo* scenario whereby loss of PKN1 increased susceptibility to I/R injury, we knocked down PKN1 expression in NRVM using si RNA, then subjected the cells to sI/R and assessed the extent of cell injury/survival. *Figure 4A* shows that under optimal conditions using two different siRNA constructs, loss of PKN1 expression was >95% compared to a scrambled sequence negative control construct. Cells were subjected to 1 h sI followed by 18 h recovery in normal maintenance medium. sI/R times were optimized to result in approx. 50% cell death in control cells as determined using MTT bioreduction as an index of cell survival. Knockdown of PKN1 had no effect on the survival of cells under control conditions. However, loss of PKN1 following siRNA knockdown resulted in a significant further loss of cell viability following sI/R (*Figure 4B*). Cell injury was assessed in parallel by measurement of creatine phosphokinase (CPK) release during the first 90 min of recovery from sI (*Figure 4C*). PKN1 knockdown resulted in an approx. doubling of CPK release during early recovery from sI. These results demonstrate that in keeping with results obtained in PKN1 KO hearts, loss of PKN1 results in increased susceptibility of cardiomyocytes to sI/R injury. 

These results demonstrate that the baseline susceptibility of cardiomyocytes to I/R injury is dependent on the PKN1 level. To directly determine an overt protective role for PKN1 in sI/R injury, we overexpressed WT-hPKN1-FLAG or KD-hPKN1-FLAG using adenoviral-mediated transduction. Western blotting against the FLAG epitope showed successful and equivalent high level expression of both species (*Figure 4D*). *In vitro* kinase assay of cell lysates following IP of WT- and KD-hPKN1-FLAG using an anti-FLAG antibody confirmed the absence of kinase activity in the KD-hPKN1 mutant (*Figure 4E*). Interestingly, both WT and KD-hPKN1 increased cell viability (*Figure 4F*) and reduced cell injury (*Figure 4G*) to an equivalent degree (approx. 30%). These results suggest that the kinase activity of PKN1 is not required for its protective function.

### 3.4 Changes in PKN1 localization during sI/R in ventricular cardiomyocytes

**Figure 5:**
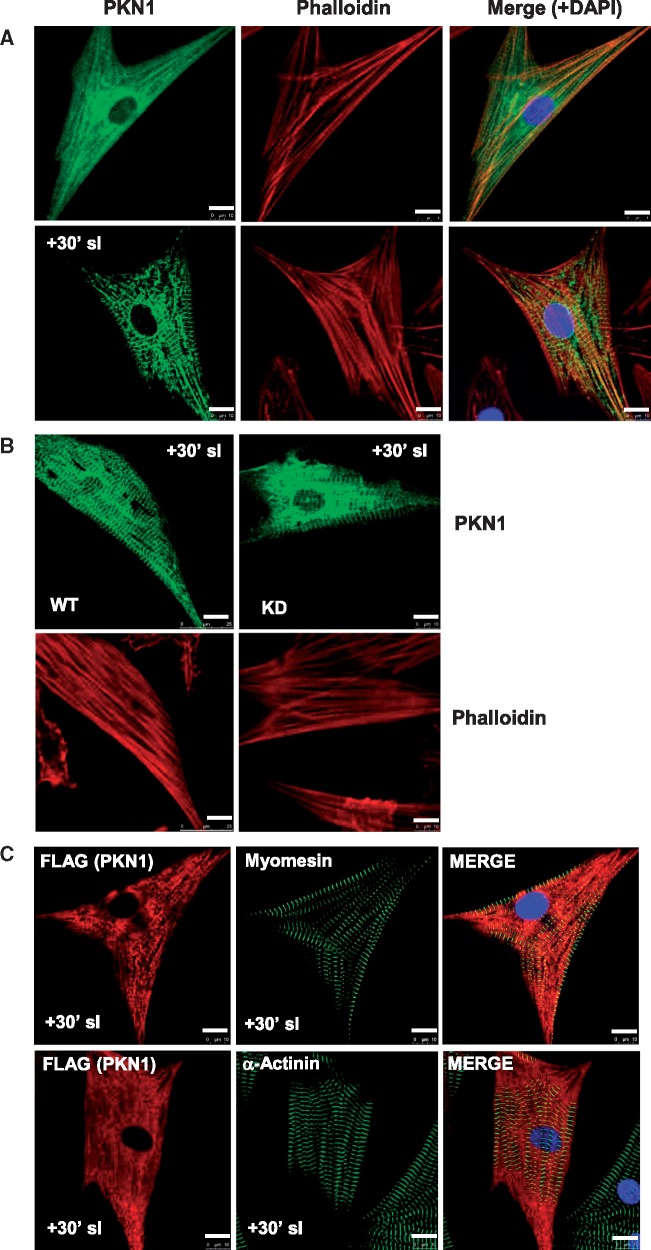
*Confocal Immunofluorescence Analysis of PKN1 Shows a Striated Redistribution During Simulated Ischaemia.* (*PanelA*) Translocation of PKN1 during SI. NRVMs infected with adenovirus expressing wild type (WT)-PKN1-FLAG and were either untreated (control) or subjected to 30 min SI prior to fixation. Slides were stained with mouse monoclonal anti-PKN1 antibody (green) and counterstained with FITC-conjugated phalloidin (red) to visualize filamentous (F)-actin and with DAPI (blue) to visualize nuclei. Slides were analysed by confocal microscopy in the separate green, red, and blue channels and a merged image is shown for the overlay of the individual images. (*PanelB*) The localization of WT-PKN1-FLAG and KD-PKN1-FLAG was compared during SI. NRVMs were infected with adenoviruses expressing WT-PKN1-FLAG (WT) or KD-PKN1-FLAG (KD) and treated with SI for 30 min prior to fixing and mounting. Slides were stained with mouse monoclonal anti-PKN1 antibody (green) and counterstained with phalloidin (red). (*PanelC*) NRVMs infected with adenovirus expressing wild type (WT)-PKN1-FLAG and were subjected to 30 min SI prior to fixation. Slides were stained with rabbit polyclonal anti-FLAG antibody (red) and counterstained with mouse monoclonal anti-myomesin antibody (green) or mouse monoclonal anti-α-actinin antibody (green), to visualize the M-band and Z-discs, respectively, and with DAPI (blue) to visualize nuclei. Slides were analysed by confocal microscopy in the separate green, red, and blue channels and a merged image is shown for the overlay of the individual images. Scale bar equals 10 μm.

We next sought to determine whether PKN1 intracellular localization changes during sI/R and whether or not PKN1 kinase activity is required for PKN1 translocation. NRVMs were transduced with FLAG-tagged hPKN1 and confocal immunofluorescence microscopy was performed using an anti-FLAG or monoclonal anti-PKN1 antibody. *Figure 5A* shows that in control cells PKN1 had a diffuse punctate staining pattern with some overlap with staining for filamentous actin (FITC-phalloidin). Interestingly, following sI PKN1 relocalized to a striated pattern with a repeating large amplitude register of approximately 1.8 μm with alternating small amplitude register consistent with a sarcomeric/myofilament localization (*Figure 5A* and *B*). Furthermore, the redistribution of WT-hPKN1-FLAG and KD-hPKN1-FLAG was identical (*Figure 5B*), suggesting that kinase activity is not required for this redistribution. 

**Figure 6:**
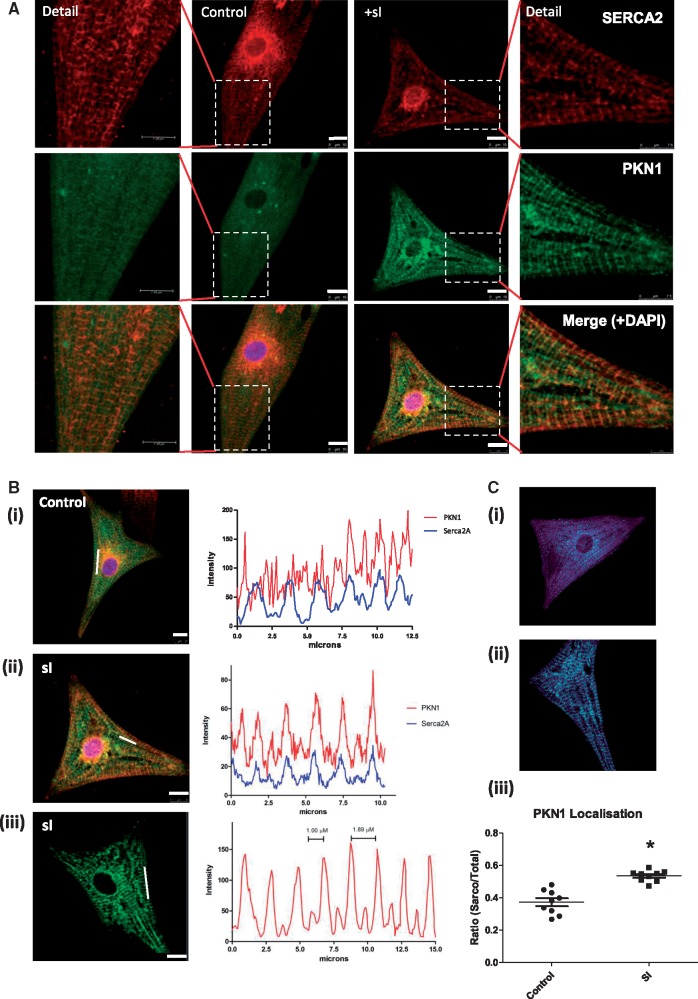
*Confocal Immunofluorescence Analysis of PKN1 Shows a Sarcoplasmic Reticulum Localization During simulated Ischaemia.* (*PanelA*) The co-localization of PKN1 and Serca2a in NRVMs was compared during SI. NRVMs were infected with adenovirus expressing WT-PKN1-FLAG and were either untreated (control) or subjected to 30 min SI prior to fixation and slide mounting. Slides were stained with mouse monoclonal anti-PKN1 antibody (green) and counterstained with Serca2a antibody to stain the SR membrane (red) and DAPI (blue). Slides were analysed by confocal microscopy in the separate green, red and blue channels and a merged image is shown for the overlay of the individual images. Scale bar is 10 μm. (*PanelB*) The coincidence of PKN1 and SERCA2 localization was determined during simulated ischaemia. Confocal images representing PKN1 in NRVMs under control conditions (i) and after 30’ SI (ii). The line charts (right panels) show the intensity plots of the Cy3 (PKN1) signal over the regions shown by a white line on the *images*. The distance between two major peaks and a major peak and minor peak are illustrated in the right panel. In (iii) the line chart shows the intensity plots of the Cy3 (PKN1) and Cy5 (SERCA2a) signals over the region shown by a white line on the merged image. In all cases, experiments were repeated four times and >10 fields analysed per treatment. (*Panel C*) Quantitation of PKN1 and SERCA2 co-localization. Panels (i) and (ii) show the masks generated for calculation of total and SERCA2 coincident PKN1. Panel (iii) shows quantitation of the fraction of SR coincident PKN1 normalized to total (cyto) cellular distributed PKN1 where *P ≤ 0.05 vs. control (*n* = 9 per group) using a nested ANOVA with three batches of cells each in triplicate for each group.

To determine whether relocalization of PKN1 corresponds to a sarcomeric redistribution, cells were co-stained for the myofilament markers myomesin (M-band) and α-actinin (z-disc). The small amplitude PKN1 band overlapped with myomesin (*Figure 5C*) and the large amplitude PKN1 band staining falls between the myomesin bands with an identical register, suggesting a myofilament localization. There was also limited overlap with α-actinin, suggesting partial Z-disc localization. Because PKN1 staining showed a sarcomeric pattern which corresponded partially to A-band or M-band and Z-disc, we compared PKN1 to the sarcoplasmic reticulum (SR) marker SERCA2a. Under control conditions staining for SERCA2 showed a striated sarcomeric pattern with some perinuclear staining consistent with ER (*Figure 6A*, top panels). Co-staining for PKN1 showed a very similar pattern (*Figure 6A*, middle panels) and the merged images show a high degree of overlap (*Figure 6A*, lower panels and *Figure 6B*). Quantitative analysis of confocal images showed a significant increase in PKN1 SR localization during sI (*Figure 6C*). 

Because of differences in SR structure and function between neonatal and adult cardiomyocytes, we next examined the relationship between PKN1 and SERCA2a in isolated adult cardiomyocytes. The results shown in Supplementary material online, *Figure S4* confirm that PKN1 also shows a high degree of co-localization with SERCA2 in adult cardiomyocytes with a fully mature and functional SR under conditions of sI. However, in control adult cardiomyocytes PKN1 showed some overlap with SERCA2a but only displayed the large amplitude register and was irregular. Following sI the pattern of PKN1 distribution was much sharper and showed the alternating large/small amplitude striated register as observed in NRVM following sI. This redistribution of PKN1 during sI in adult cardiomyocytes is represented schematically in Supplementary material online, *Figure S5*. Interestingly, the resident ER protein reticulon 4 (NogoA) also co-localized with PKN1 under these conditions. See Supplementary material online, *Figure S6* shows that in sections of normal adult mouse hearts PKN1 immunofluorescence has a striated pattern coincident with phalloidin staining of actin at the I-band. However, this would also be adjacent to SERCA2 at the SR (see also see Supplementary material online, *Figure S5*). Taken together, these results show that PKN1 localization overlaps with SERCA2 at the SR and that this localization is enhanced during sI, suggesting a role for PKN1 in regulating SR function during ischaemia.

### 3.5 Interaction of PKN1 with ER/SR-associated proteins

We reasoned that PKN1 relocalization to SR during ischaemia may be related to its interaction with other resident SR/ER proteins which are linked to its protective function. Therefore, to gain further insights into possible interacting partners, NRVMs were transduced with adenoviruses expressing hPKN1-FLAG and treated with sI for 30 min. hPKN1 was harvested by immunoprecipitation using an anti-FLAG Ab and candidate binding partners were identified by mass spectrophotometric analysis. Using this approach PKN1 was found to be associated with several resident ER/SR proteins only during sI. These included reticulon 4 (Nogo A), calreticulin, reticulocalbins 1 and 2 (RCN1 and 2), as well as 14-3-3γ and the E3 ubiquitin ligase NEDD4 (see Supplementary material online, *Table ST1*). In addition, PKN1 associated with Ca^2+^-calmodulin-dependent kinase 2 delta (CamKIIδ) which is a regulator of SR Ca^2+^ loading and release via SERCA2 and the ryanodine receptor (RyR2). Binding to 14-3-3γ was confirmed in cell lysates only following sI (see Supplementary material online, *Figure S7*).

**Figure 7:**
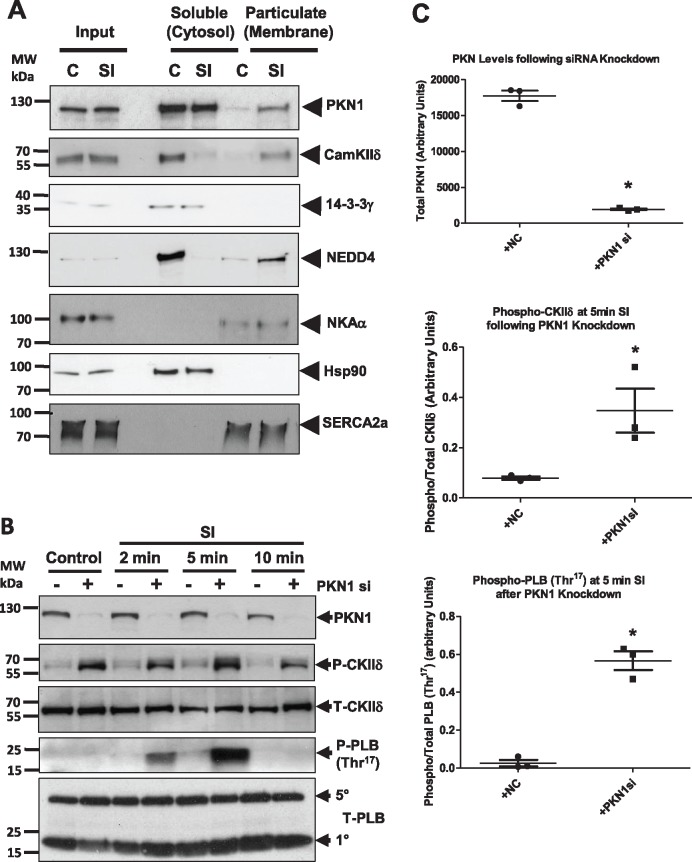
*Association of PKN1 with Sarco-Endoplasmic Reticulum Binding Partners Correlates with CamKIIδ and Phospholamban Thr* ^17^ *Phosphorylation During Simulated Ischaemia.* (*PanelA*) NRVM were either untreated or subjected to sI and fractionated into soluble and membrane fractions. Fractions were subjected to SDS-PAGE and Western blotting and probed for PKN1 and its binding partners CamKIIδ, 14-3-3γ, and NEDD4 or compartmental markers Na/K-ATPase (sarcolemmal membrane), Hsp90 (cytosol), and SERCA2a (SR membrane). (*PanelB*) NRVM in which PKN1 was knocked down using siRNA were compared to control cells at different times of simulated ischaemia (sI) and analysed for phosphorylation of CamKIIδ (Thr^287^) or phospholamban (PLB: Thr^17^). For all panels representative images are shown for one of three independent experiments. (*PanelC*) Quantitation of PKN1, phosphor-CamKIIδ (Thr^287^) and phosphor-PLB (Thr^17^) at 5 min of sI following treatment with negative control (+NC) siRNA or PKN1 siRNA (+PKN1si). Phosphor-CamKIIδ and phosphor-PLB were normalized to total CamKIIδ and total PLB, respectively. *P ≤ 0.05 or **P ≤ 0.001 (*n* = 3) unpaired students *t*-test.

In order to explore these interactions further we exposed cells to sI/R, fractionated them into soluble and particulate (membrane) fractions and probed the fractions for putative partners and compartmental markers. *Figure 7A* shows that PKN1 levels increased in the particulate fraction in parallel with a marked redistribution of CamKIIδ and NEDD4. Strict partitioning of Na^+^/K^+^ ATPase (NKA) α subunit and SERCA2 to the particulate fraction and Hsp90 to the soluble fraction demonstrate the fidelity of the fractions and also that the particulate fraction contains both sarcolemmal and SR membranes. 

We next determined whether during sI, PKN1 affects the phosphorylation of CamKIIδ and its downstream SR target phospholamban (PLB), an endogenous regulator of SERCA2 activity. Endogenous PKN1 expression was either left intact or knocked down using siRNA in NRVMs prior to exposure to sI for various times. The results show that under control conditions and during sI, loss of PKN caused a dramatic increase in CamKIIδ Thr^287^ phosphorylation indicative of kinase activation (*Figure 7B*). Furthermore, there was a marked increase in CamKIIδ-dependent PLB Thr^17^ phosphorylation at 2–5 min. By 10 min sI PLB Thr^17^ phoshorylation returned to baseline. These results indicate that loss of PKN1 results in a marked activation of CamKIIδ and downstream PLB Thr^17^ phosphorylation.

**Figure 8:**
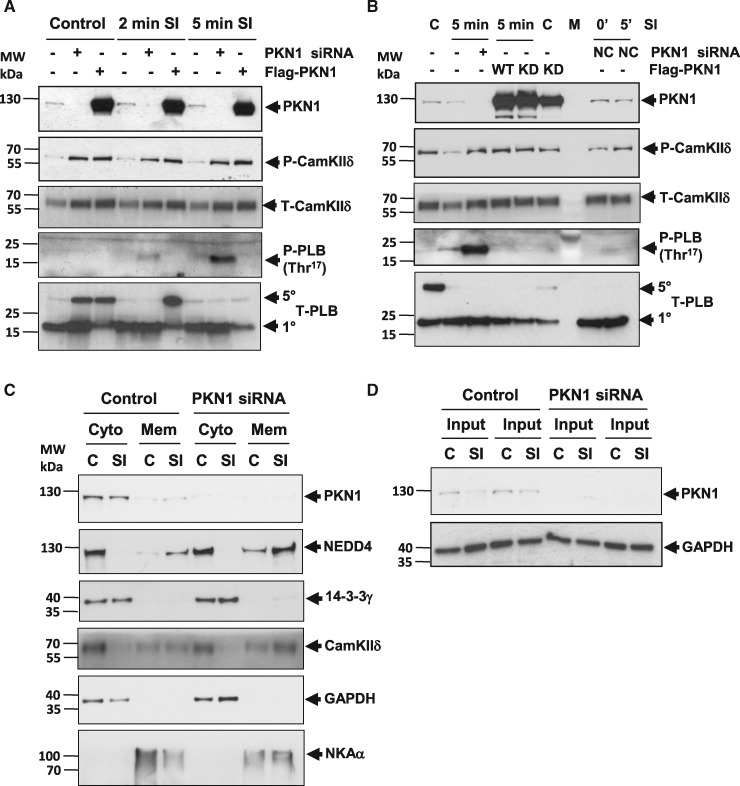
*CamKIIδ and Phospholamban Thr* ^17^ *Phosphorylation Are increased Following Knockdown of PKN1*. (*Panel A*) NRVM in which PKN1 was either knocked down using siRNA or overexpressed (Flag-PKN1) were compared to control cells at 2 min or 5 min of simulated ischaemia (sI) and analysed for phosphorylation of CamKIIδ (Thr^287^) or phospholamban (PLB: Thr^17^). (*Panel B*) NRVM overexpression of wild type (WT) or kinase dead K^644^R (KD) PKN1 were compared at 5 min of simulated ischaemia (sI) and analysed for phosphorylation of CamKIIδ (Thr^287^) or phospholamban (PLB: Thr^17^). (*Panel C*) NRVM in which PKN1 was knocked down using siRNA were treated with sI/R and fractionated into soluble and membrane and then analysed for binding partners CamKIIδ, 14-3-3γ and NEDD4 or compartmental markers Na/K-ATPase (sarcolemmal membrane), Hsp90 (cytosol), and SERCA2a (SR membrane). (*Panel D*) Input material for comparison or protein loading. For all panels, representative images are shown for one of three independent experiments.

We then compared the effect of siRNA knockdown of PKN1 to overexpression of PKN1 on CamKIIδ activation and PLB phosphorylation. PLB phosphorylation increased during 2–5 min sI only following PKN1 knockdown (*Figure 8A*), whereas in the presence of overexpressed WT-hPKN1 PLB phosphorylation was absent (*Figure 8B*). There was also no change in PLB phosphorylation following overexpression of KD-hPKN1 suggesting that inhibition of CamKIIδ-dependent PLB phosphorylation is independent of PKN1 kinase activity. 

To investigate the effect of PKN1 knockdown on CamKIIδ and NEDD4 redistribution cells were exposed to sI following PKN1 siRNA treatment and fractionated. NEDD4 localization to the particulate (membrane) fraction was increased by PKN1 knockdown. CamKIIδ redistribution to the membrane fraction was also increased upon loss of PKN1 (*Figure 8C*). Input material is shown in *Figure 8D*.

### 3.6 Effect of PKN1 loss on SR Ca^2+^ handling proteins *in vivo*

**Figure 9:**
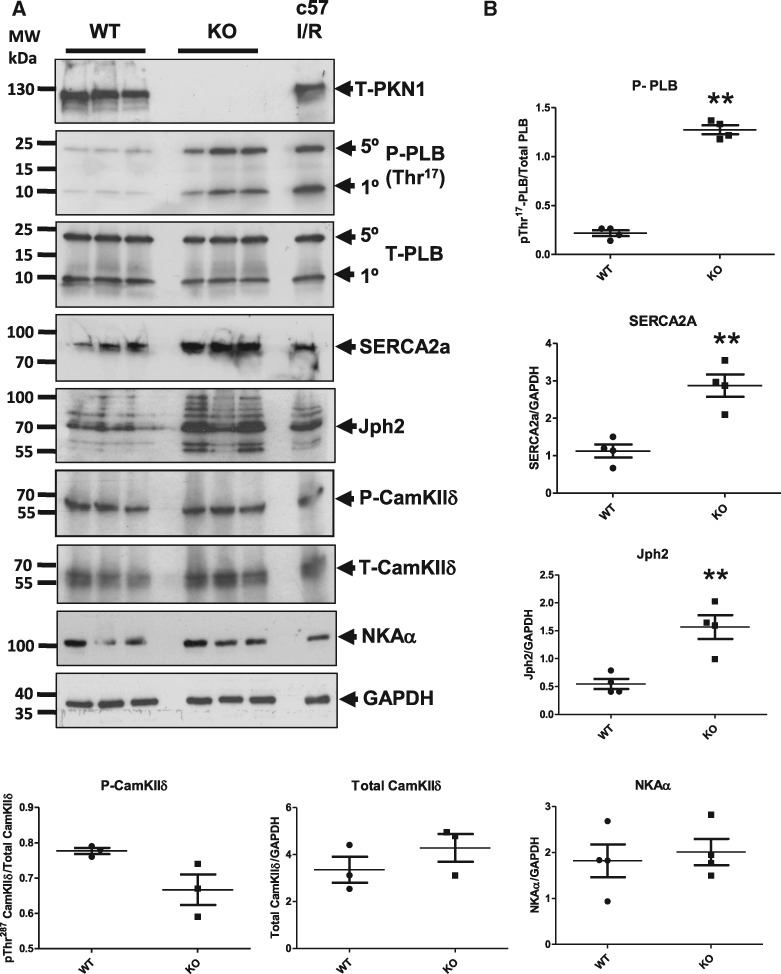
*Phospholamban Thr* ^17^ *, SERCA2a and Junctophilin-2 Levels are Increased in PKN1 Knockout Hearts.* Hearts were isolated from wild type (WT) and PKN1 knockout (KO) mice, subjected to SDS-PAGE and Western blotting. (*Panel A*) Total PKN1, phospho- (Thr^17^) and total phospholamban (PLB), SERCA2a, phospho- (Thr^287^) and total CamKIIδ, junctophilin-2 (Jph2) and the Na^+^/K^+^ ATPase (NKAα). GAPDH was used as a loading control. (*Panel B*) Quantitative analysis of levels and statistical analysis using an unpaired *t*-test where **P ≤ 0.01. Proteins were quantitated by densitometry and normalized to total GAPDH and compared using a two-tailed unpaired *t*-test (*n* = 4 individual hearts). Each sample represents a different lysate prepared from an individual heart.

We next investigated whether the loss of PKN1 resulted in altered SR protein levels or phosphorylation *in vivo*. Hearts were isolated from WT and PKN1 KO mice at baseline. *Figure 9* shows that basal phosphorylation of PLB Thr^17^ was greatly increased in PKN1 KO hearts compared to WT whereas levels of total PLB were unchanged. The increase in PLB Thr^17^ phosphorylation in PKN1 KO hearts was equivalent to the level of phosphorylation observed in c57Bl/6 hearts during I/R. Interestingly, levels of SERCA2a were also increased in PKN1 KO hearts. Since PLB phosphorylation de-represses SERCA2 activity, together these results suggest a compensatory enhancement of SR Ca^2+^ uptake in PKN1 KO hearts. Levels of CamKIIδ phosphorylation and expression were unchanged at baseline. Levels of the Na^+^/K^+^ ATPase (NKA) were also unaffected. Furthermore, increased expression of the junctional membrane complex (JMC) structural component Junctophilin-2 (Jph2) was also observed (*Figure 9*). 

### 3.7 Functional changes in PKN1 KO hearts

**Figure 10:**
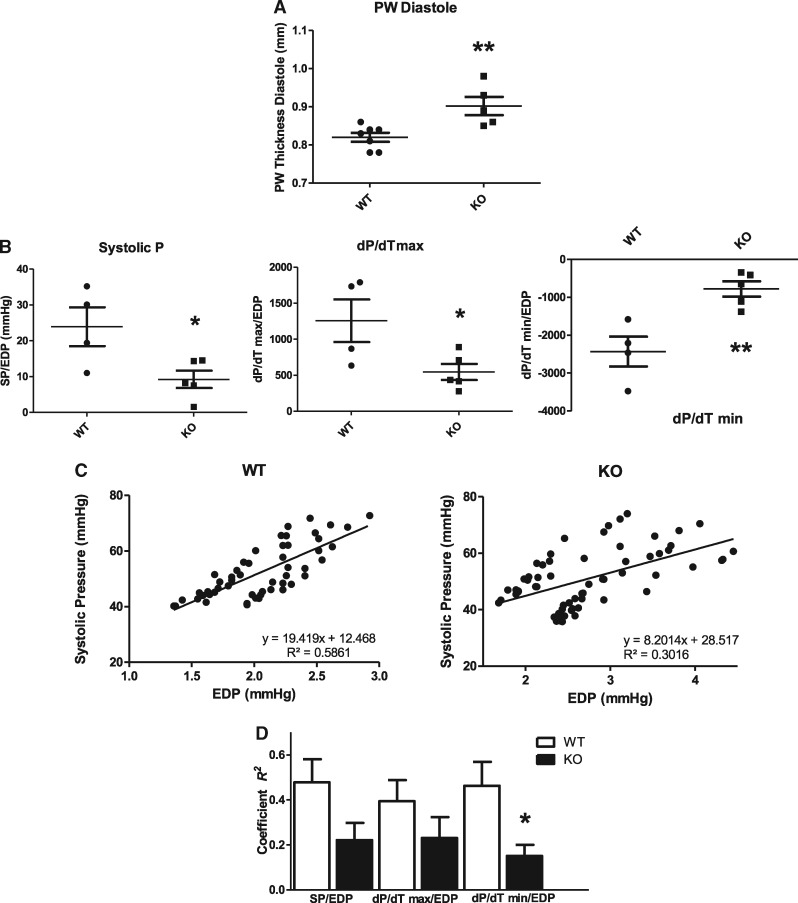
*Echocardiographic and P-V Loop Analysis of Cardiac Contractile Function Shows Mild Diastolic Dysfunction in PKN1 KO Hearts.* (*Panel A*) Differences in left ventricular (LV) posterior wall thickness at diastole (PWTD) between wild type (WT: *n* = 7) and knockout (KO: *n* = 5) mice derived from Doppler echocardiography. (*Panel B*) Relationships between systolic pressure (SP), maximal rate of contraction (dP/dT max) and maximal rate of relaxation (dP/dT min) and end diastolic pressure (EDP) in WT (*n* = 4) and PKN1 KO (*n* = 5) hearts derived from PV loop analysis. (*Panel C*) Correlation of beat-to-beat differences in LV SP vs. EDP for a single WT and PKN1 KO mouse derived from PV loop analysis. (*Panel D*) Comparison of correlation coefficients (*R*^2^) for SP/EDP, dP/dT max/EDP, and dP/dT min/EDP for WT (*n* = 4) and PKN1 KO (*n* = 5) mice derived from PV loop analysis.

These molecular changes in SR Ca^2+^ regulatory proteins would be expected to result in altered SR Ca^2+^ handling and thus be reflected by changes in cardiac contractile performance. In order to test this possibility, WT and PKN1 KO hearts were subjected to echocardiographic and pressure-volume (PV) loop analysis using an admittance catheter inserted into the left ventricle. The results shown in Supplementary material online, *Figure S8* show that PKN1 KO hearts were essentially compensated at baseline and had normal ejection fraction, systolic and diastolic volumes and pre-load-independent relaxation constant (Tau). Normal systolic and diastolic volumes and anterior wall thickness were also confirmed by echocardiographic analysis (results not shown). However, posterior wall thickness at diastole (PWTD) was significantly higher in KO hearts (*Figure 10A*). Furthermore, PKN1 KO hearts had a trend towards altered functional characteristics compared to wild type hearts under conditions of decreased preload (IVC occlusion), including decreased end systolic pressure-volume relationship (ESPVR), increased end diastolic pressure-volume relationship (EDPVR) and decreased preload recruitable stroke work (PRSW), although these changes did not reach statistical significance (see Supplementary material online, *Figure S8*). However, more detailed analysis of the beat-to-beat relationship between systolic pressure (SP), the maximal rate of systolic pressure development (dP/dT max) and the maximal rate of relaxation (dP/dT min) normalized to the end diastolic pressure (EDP) of the preceding cycle showed a significant decrease in systolic P and also a decrease in the maximal rate of systolic P generation and the rate of relaxation in KO hearts (*Figure 10B*). This is suggestive of an underlying systolic and diastolic dysfunction with preserved ejection fraction (EF: see Supplementary material online, *Figure S8*) which relates to an altered Frank-Starling response. Representative scatter plots of SP vs. EDP for an individual WT and KO mouse are shown in *Figure 10C*. Intra-beat relationships for EDP vs. SP, EDP vs. rate of contraction (dP/dT max) and EDP vs. rate of relaxation (dP/dT min) were determined. The intra-beat relationships between EDP and SP, EDP and dP/dT max (+dP/dT) and EDP and dP/dT min (-dP/dT) determined as the coefficient (*R^2^*) were significantly decreased in KO hearts (*Figure 10D*) indicating that coupling between EDP and contractile function was significantly impaired in PKN1 KO hearts. 

## 4. Discussion

The results obtained in this study show that loss of PKN1 increases susceptibility to ischaemia/reperfusion (I/R) injury both in the intact heart and in isolated, cultured cardiomyocytes, suggesting a cell-autonomous, intrinsic basal cardioprotective role for PKN1 in cardiomyocytes and as previously suggested.^24^ Loss of PKN1 was associated with increased activity of CamKIIδ and phosphorylation of its downstream substrate PLB on Thr^17^ as well as upregulation of the sarcoplasmic reticulum ATPase SERCA2. These results suggest that PKN1 has a scaffold role which may function to prevent or limit CamKIIδ access to specific substrates such as PLB during ischaemia. Translocation of PKN1 and association with CamKIIδ at the SR during sI correlates with PKN1-dependent cardiomyocyte cytoprotection. Since PKN1-dependent cardioprotection is intrinsic to cardiomyocytes, the model of global ischaemia used in this study is consistent with results from *in vivo* studies.^24^

In freshly isolated spontaneously contracting NRVM the SR is reportedly less well developed and excitation contraction coupling is mainly dependent on extrinsic Ca^2+^ entry via L-type voltage-gated Ca^2+^ channels. However, after several days in culture the SR in NRVM was distinct and well developed and involved in Ca^2+^ sequestration and release.^25^^,^^27^^,^^28^ The cellular distribution of PKN1 was similar in NRVMs and adult cardiomyocytes and in both cases the tight alternating large/small amplitude double striated register only appeared under (simulated) ischaemic conditions. Irrespective of developmental differences in SR structure/function, loss of PKN1 was associated with an increase in PLB Thr^17^ phosphorylation both in adult hearts and NRVM. Therefore, taken together these results suggest that the presence of PKN1 at the SR itself inhibits CamKIIδ-dependent PLB phosphorylation.

Dysregulation of SR Ca^2+^ handling during reperfusion following ischaemia correlates with CamKIIδ activity^29–32^ and CamkIIδ has an established role in promoting I/R injury since inhibition of CamKIIδ limits infarct size.^33–35^ Cytosolic Ca^2+^ overload during I/R is associated with cytosolic Ca^2+^ oscillations which occur prior to cardiomyocyte cell death due to mitochondrial Ca^2+^ overload and MTP pore opening.^36^ These Ca^2+^ oscillations are due to increased SR Ca^2+^ loading via increased SERCA2 activity (ie following PLB Ser^16^/Thr^17^ phosphorylation) coupled with Ca^2+^ leak via the SR Ca^2+^ release channel (ryanodine receptor: RyR2). RyR2 destabilization or increased open probability during I/R has been attributed to a number of mechanisms including Ser^2814^ phosphorylation by CamKIIδ itself, loss of calstabin1 (FKBP12.6) binding and Ca^2+^/calpain-dependent proteolytic degradation of RyR2.^37^ We did not observed any direct evidence of RyR Ser^2814^ phosphorylation (results not shown). However, it is also possible that destabilization of the T-tubule/SR junctional membrane complex (JMC) may occur due to Ca^2+^-dependent degradation of other key structural proteins such as junctophilin-2 (Jph2) which stabilizes the JMC and is degraded following I/R.^38^^,^^39^ We observed increased expression of Jph2 in PKN1 KO hearts (*Figure 9*) suggesting turnover and/or remodelling of JMC components. Although speculative without further detailed analysis of SR function, these results suggest that coupling of SR Ca^2+^ handling is destabilized in PKN1 KO hearts which could increase Ca^2+^ overload during I/R. The main events involved in cardiomyocyte cytosolic Ca^2+^ overload following I/R are summarized schematically in Supplementary material online, *Figure S9*.

PLB Thr^17^ phosphorylation relieves its inhibitory action on SERCA2, thus increasing SERCA2 activity. Coupled with increased SERCA2 levels in PKN1 KO hearts this would be expected to be associated with increased SR Ca^2+^ loading and faster relaxation or recovery of relaxation following I/R.^40^ This in itself may be protective in I/R provided that it is not accompanied by increased SR leak. Increased SR Ca^2+^ loading would be reflected by increased myocardial contractility as indicated by increased dP/dT_max_ and ESPVR. This was not observed in PKN1 KO hearts. In fact PKN1 KO hearts had the same average dP/dT_max_ and ejection fraction as WT hearts. However, beat-to-beat systolic P and dP/dT max normalized to EDP were significantly decreased in KO hearts as was dP/dT min, whereas diastolic posterior wall thickness was increased, suggesting increased diastolic stiffness and decreased systolic function relative to changes in EDP which may be due to increased resting cytosolic Ca^2+^. Therefore, it is likely that stroke volume (SV) and systolic P (SP) are compensated in KO hearts at the expense of elevated EDP. Interestingly, the phenotype of the PKN1 KO mice is strikingly similar to protein kinase G Iα (PKGIα) Cys^42^Ser knock-in mice which centres on oxidation-dependent alterations in PLB (Ser^16^) phosphorylation and the coupling of EDP changes to contractile function.^41^ However, since EDP was not significantly different during reperfusion following ischaemia [*Figure 1A* (*ii*)], increased SR Ca^2+^ loading may mitigate against increased EDP during reperfusion.

Whilst these changes could reflect defective SR function and increased cytosolic diastolic Ca^2+^, it could also be postulated based on the increased SERCA2a levels and PLB Thr^17^ phosphorylation that higher SR Ca^2+^ load and PLB Thr^17^ phosphorylation may be expected increase the frequency and amplitude of cytosolic Ca^2+^ oscillations following IR injury^36^ and spontaneous SR Ca^2+^ release (Ca^2+^ spark) frequency at baseline thus exacerbating I/R injury. This is supported since delayed PLB phosphorylation contributes to the protection afforded by ischaemic post-conditioning^42^ and although PLB ablation rescues SR Ca^2+^ loading, it exacerbates injury.^30^ Alternatively, the elevation of SERCA2a and Jph2 expression and PLB Thr^17^ phosphorylation may be an abortive adaptive response to dysregulated SR Ca^2+^ handling in PKN1 KO hearts (ie SR Ca^2+^ leak). These possibilities need to be explored further.

PKN1 Thr^774^ phosphorylation in the activation loop by PDK1 has been shown to be essential for PKN1 catalytic activity and stability.^14–16^ Although modest Thr^778^ phosphorylation was observed during I/R in intact hearts and sI/R in isolated, cultured NRVM, Thr^774^ phosphorylation of ectopically expressed kinase dead (KD) PKN1 was also observed. Furthermore, the kinase activity of PKN1 was not required for its protective effect because ectopic expression of KD(K^644^R)-hPKN1 was as protective as wild type hPKN1 against sI/R in NRVM. Irrespective of Thr^774^ phosphorylation, these results suggest the protective role of PKN1 is due to a scaffold/assembly function not directly dependent on downstream substrate phosphorylation by PKN1.

Another recent example of a kinase-independent endogenous cardioprotective function has been described for PI3Kγ^43^ which, like PKN1 in this instance, behaves as a pseudokinase. Although PI3Kγ is reported to be responsible for Reperfusion Injury Salvage kinase (RISK) pathway (Akt/ERK1/2) activation downstream of GPCR activation during reperfusion,^5^ intriguingly a kinase dead PI3Kγ^KD/KD^ knock-in onto the PI3Kγ KO background rescued the PI3Kγ KO phenotype. Furthermore, PI3Kγ KO was associated with decreased ERK1/2 activation and increased basal PLB Ser^16^ phosphorylation which is the adjacent cAMP-dependent (PKA) site.^43^ CamKIIδ Thr^17^ phosphorylation wasn’t determined in their study, but is also likely to be increased. Intriguingly p42/p44-MAPK (ERK1/2) activation was also decreased in PKN1 KO hearts at baseline and following I/R and may contribute to the loss of cardioprotection. However, this possibility needs to explored directly. Therefore, the PI3Kγ KO and PKN1 KO mice have strikingly similar phenotypes. Also, RCN1 has been shown to act as an inhibitor of the activation of the B-Raf-MEK-ERK pathway in cardiomyocytes via interaction with B-Raf.^44^ The inhibition of B-Raf by RCN1 is Ca^2+^-dependent. Therefore, because we observed an interaction between PKN1 and RCN1, PKN1 could activate ERK signalling during I/R by interaction with and inhibition of RCN1 which impinges on Ca^2+^ regulation at the level of PLB phosphorylation at the SR. This would be consistent with a loss of PKN1 resulting in reduced ERK activation.

Analysis of global PKN1 phosphorylation (i.e. on sites other than Thr^778^) during sI/R using gels containing PhosTag™ reagent showed a net dephosphorylation of PKN1. This was confirmed by mass spectrometry (results not shown) with a loss of phosphorylation on 9 and a gain of phosphorylation on 6 residues (out of 20) resulting in a net loss of 3. These results show that PKN1 phosphorylation is highly dynamic during sI/R. The roles of sites other than Thr^774/778^ are not known in detail. However, sites within the N-terminal regulatory domain and linker region may be involved in PKN1 localization.^17^ Also, a site or sites in the C-terminal kinase domain may be required for PKN1 localization to a late endosomal compartment^18^ since truncated PKN1 consisting of only the regulatory domain did not localize to this compartment. However, a kinase dead PKN1 mutant localized in response to hyperosmotic stress as for wild type, but accumulated to high levels, suggesting that PKN1 kinase activity is required for exit from this compartment.^18^ Further detailed analysis will be required to determine the role of additional phosphorylation sites in PKN1. In this study, we have not specifically addressed the role of small GTPases (Rho/Rac) in PKN1 protective function. However, given that kinase activity does not appear to be important, it seems unlikely that the Rho/Rac-PDK1-PKN1 axis plays a prominent role. However this cannot be entirely excluded because the KD(K^644^R)-hPKN1 mutant was still phosphorylated on Thr^774^ by upstream signals.

PKN1 kinase activity not playing a facilitative or essential role in protection is somewhat divergent from the findings of Takagi *et al*.^24^ Whilst these authors demonstrated that cardiomyocyte-specific transgenic overexpression of constitutively active PKN1 was protective (as we found for wild type PKN1), transgenic overexpression of a dominant negative, kinase dead K^644^D mutant exacerbated I/R injury, rather than also protecting. K^644^ is a conserved lysine in the ATP binding pocket and is essential for ATP binding and activity. K^644^R substitution is a classic strategy for generating kinase-dead mutants and maintains the overall size and charge distribution in the ATP binding pocket and is therefore assumed not to have other effects on structure, whereas the introduction of an aspartate (i.e. as in K^644^D) introduces a negative charge in place of a positive charge. This may have additional effects on structure or accessory protein/substrate binding etc. In our hands, K^644^R-PKN1 is clearly not dominant negative with respect to the protective function of PKN1.

Mass spectrophotometric analysis of PKN1 immunoprecipitates following sI (30 min) demonstrated PKN1 association with several sarco-endoplasmic reticulum proteins including reticulon 4 (NogoA), calreticulin, reticulocalbin 1 (RCN1), and reticulocalbin 2 (RCN2) consistent with translocation to SR membranes. Interestingly, PKN1 also showed association with 14-3-3γ and the E3 ubiquitin ligase NEDD4 (see Supplementary material online, *Table ST1*) as well as CamKIIδ. Cell fractionation studies confirmed that during sI PKN1 co-localized to the membrane fraction with CamkIIδ and NEDD4, whereas 14-3-3γ did not translocate (*Figure 8A*). Following siRNA knockdown of PKN1 translocation of NEDD4 and CamKIIδ were increased (*Figure 9C*). This correlates with increased CamKIIδ activity and PLB Thr^17^ phosphorylation under the same conditions (*Figure 7B*). Interestingly, overexpression of WT- or KD-hPKN1 inhibited Thr^17^ phosphorylation without affecting CamKIIδ Thr^287^ phosphorylation. This suggests that the predominant effect of PKN1 is to prevent CamKIIδ-dependent PLB Thr^17^ phosphorylation rather than upstream CamKIIδ phosphorylation itself. Since there is no global inhibition of CamKIIδ (Thr^287^ phosphorylation), this is likely to be a localized effect at the SR membrane and may be a function of PKN1-dependent release of CamKIIδ from the membrane. The role of PKN1 interaction with NEDD4 is unknown. However, Takagi *et al*.^24^ attributed PKN1 protective function to facilitation of ubiquitin-proteasomal protein degradation. Despite this, we found no effect of proteasome inhibitors on the protective effect of PKN1 in cardiomyocytes (result not shown). Interaction of PKN1 with 14-3-3γ in heart has been shown previously, where PKN1 is recruited to a large signalling complex by A-kinase anchoring protein AKAP-Lbc, recruiting and activating p38α-MAPK. The complex is negatively regulated by 14-3-3γ.^45^^,^^46^ This may be relevant given the known role of p38α-MAPK in ischaemic injury, however, we found no effect of PKN1 knockout or knockdown on p38α-MAPK activation.

In conclusion, PKN1 is shown to provide a basal cardioprotective function in the face of I/R injury. This protection is intrinsic to cardiomyocytes and involves attenuation of CamKIIδ-dependent phosphorylation events at the SR and possible stabilization of the junctional membrane complex and thus SR Ca^2+^ uptake/release mechanisms and susceptibility to Ca^2+^ overload at reperfusion.

## 

**Conflict of interest**: none declared.

## Funding

This work was supported by project grant #PG/10/045/28276 from the British Heart Foundation.

## Supplementary Material

cvx206_Online_Supplementary_Figures

cvx206_Online_Supplementary_Information

## Notes

Time for primary review: 36 days
