# Supplementary material for: Loss of Protein Kinase Novel 1 (PKN1) is associated with mild systolic and diastolic contractile dysfunction, increased phospholamban Thr17 phosphorylation, and exacerbated ischaemia-reperfusion injury
Source: Cardiovasc Res. 2017 Oct 16;114(1):138–57. doi: 10.1093/cvr/cvx206 (PMC5815577; doi:10.1093/cvr/cvx206)
Supplement: cvx206_Online_Supplementary_Figures [file cvx206_online_supplementary_figures.pdf]

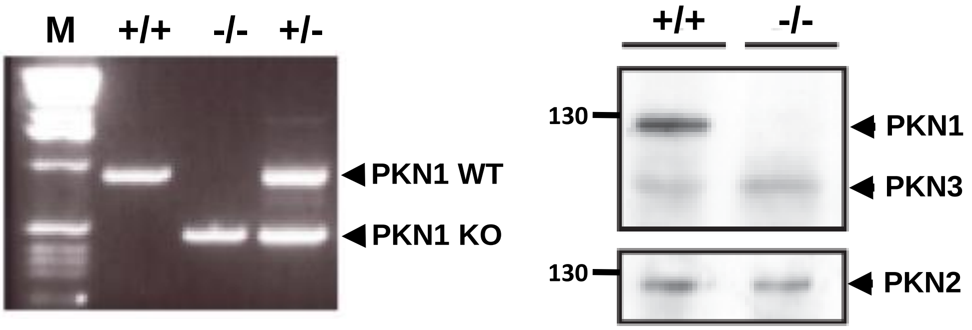

Figure S1

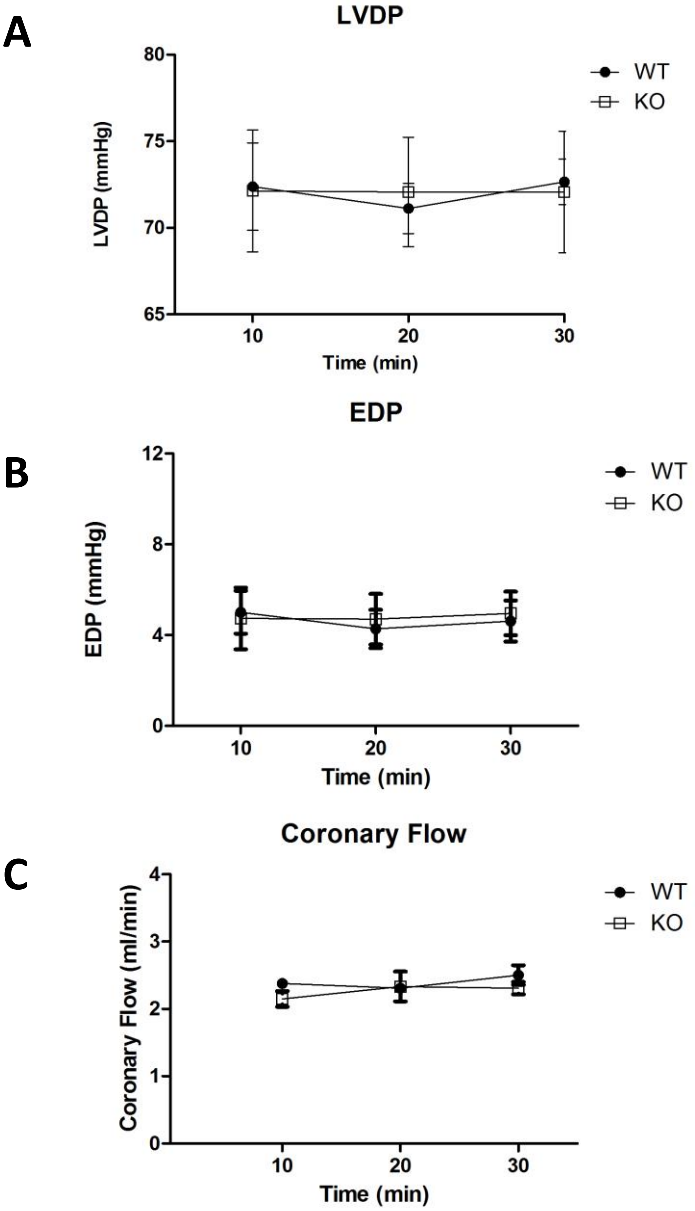
A
LVDP
80
75
70
65
LVDP (mmHg)
10
20
30
Time (min)
WT
KO
B
EDP
12
8
4
0
EDP (mmHg)
10
20
30
Time (min)
WT
KO
C
Coronary Flow
4
3
2
1
0
Coronary Flow (ml/min)
10
20
30
Time (min)
WT
KO

Figure S2

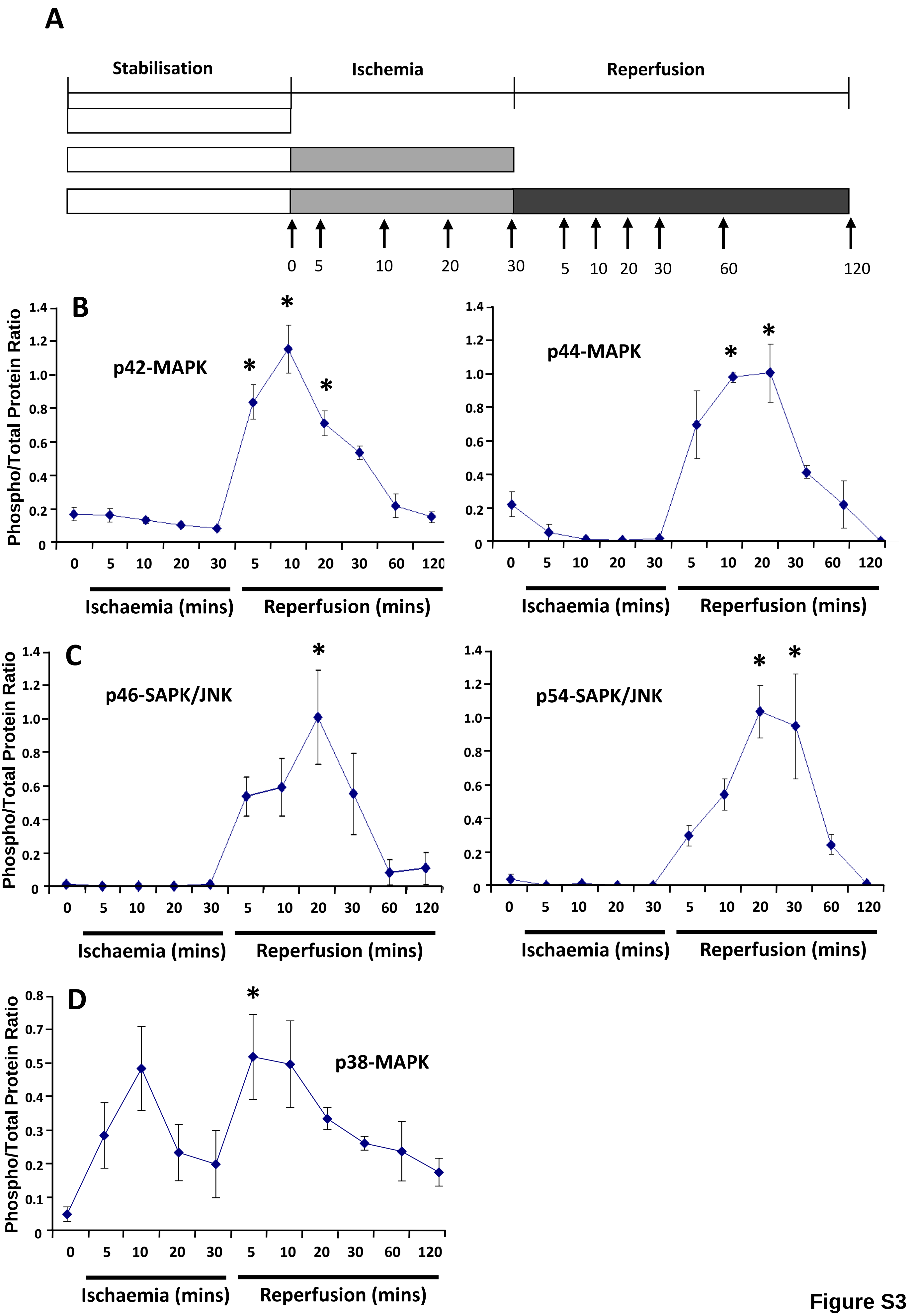
A
Stabilisation
Ischemia
Reperfusion
0 5 10 20 30 5 10 20 30 60 120
B
p42-MAPK
p44-MAPK
C
p46-SAPK/JNK
p54-SAPK/JNK
D
p38-MAPK
Phospho/Total Protein Ratio
Ischaemia (mins)
Reperfusion (mins)

Figure S3

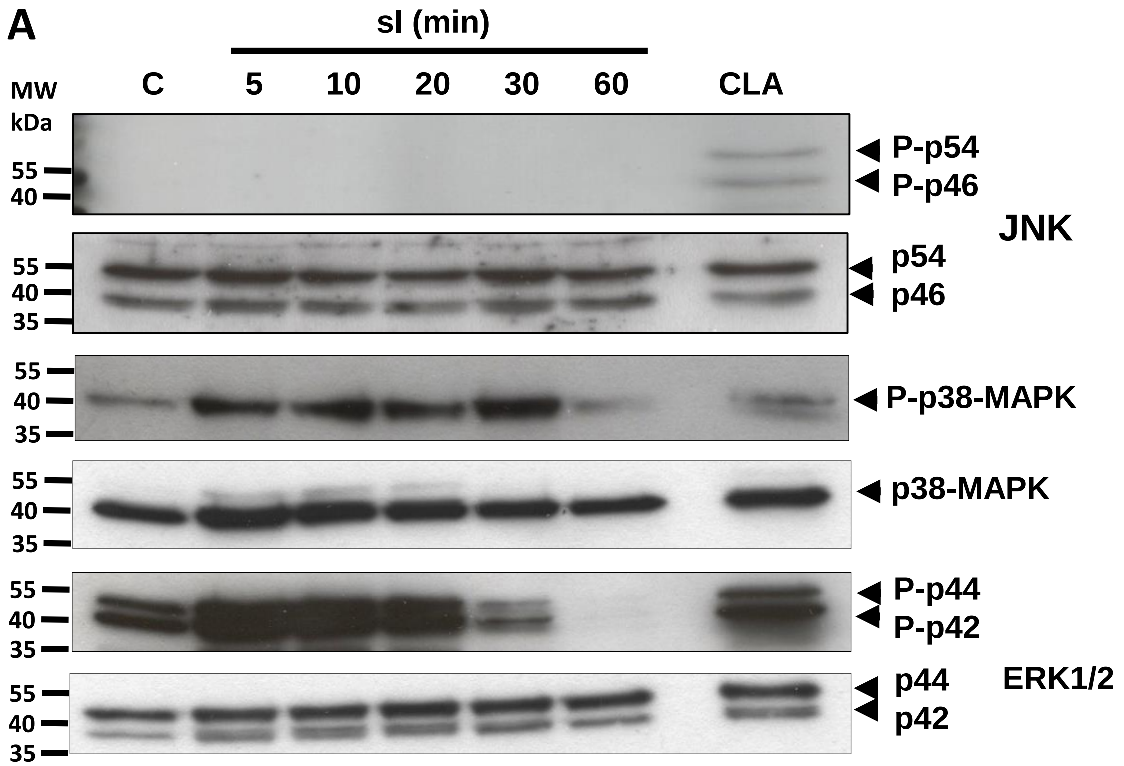

A
sI (min)
MW kDa
C
5
10
20
30
60
CLA
55
40
35
P-p54
P-p46
JNK
p54
p46
P-p38-MAPK
p38-MAPK
P-p44
P-p42
p44
p42
ERK1/2

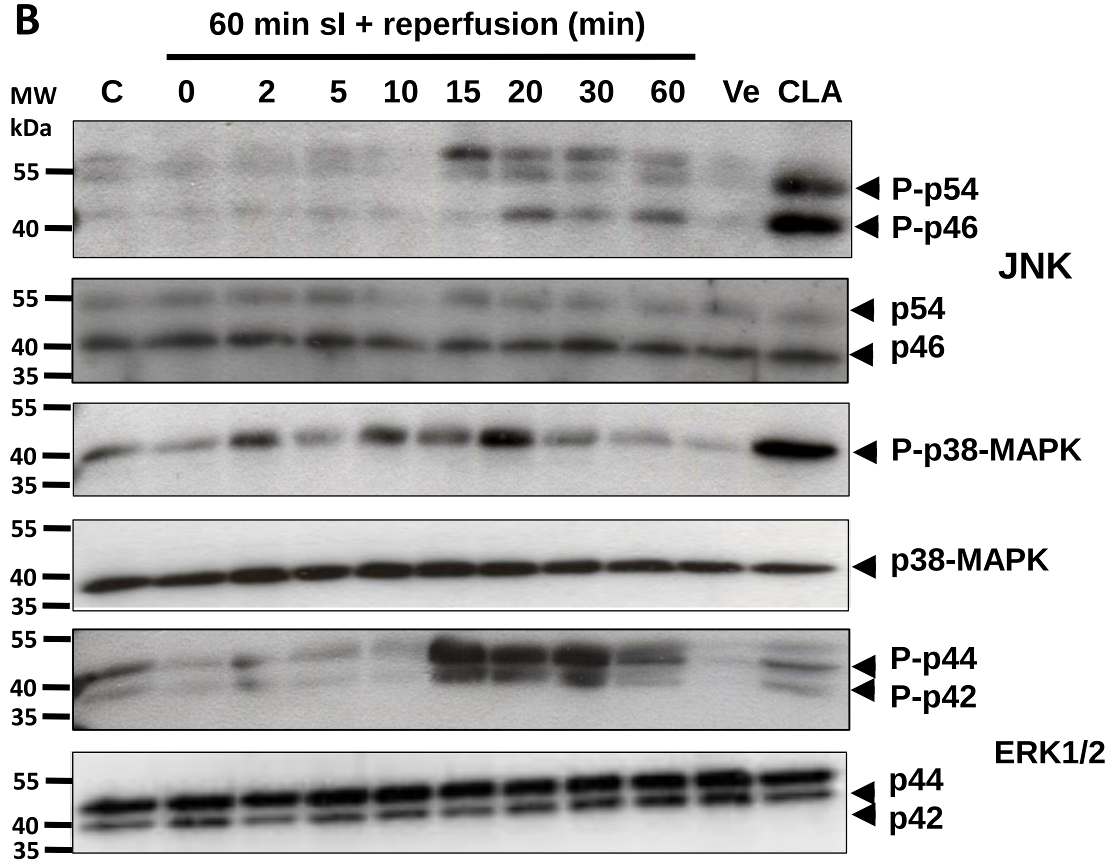

B
60 min sI + reperfusion (min)
MW kDa
C
0
2
5
10
15
20
30
60
Ve
CLA
55
40
35
P-p54
P-p46
JNK
p54
p46
P-p38-MAPK
p38-MAPK
P-p44
P-p42
p44
p42
ERK1/2

Figure S4

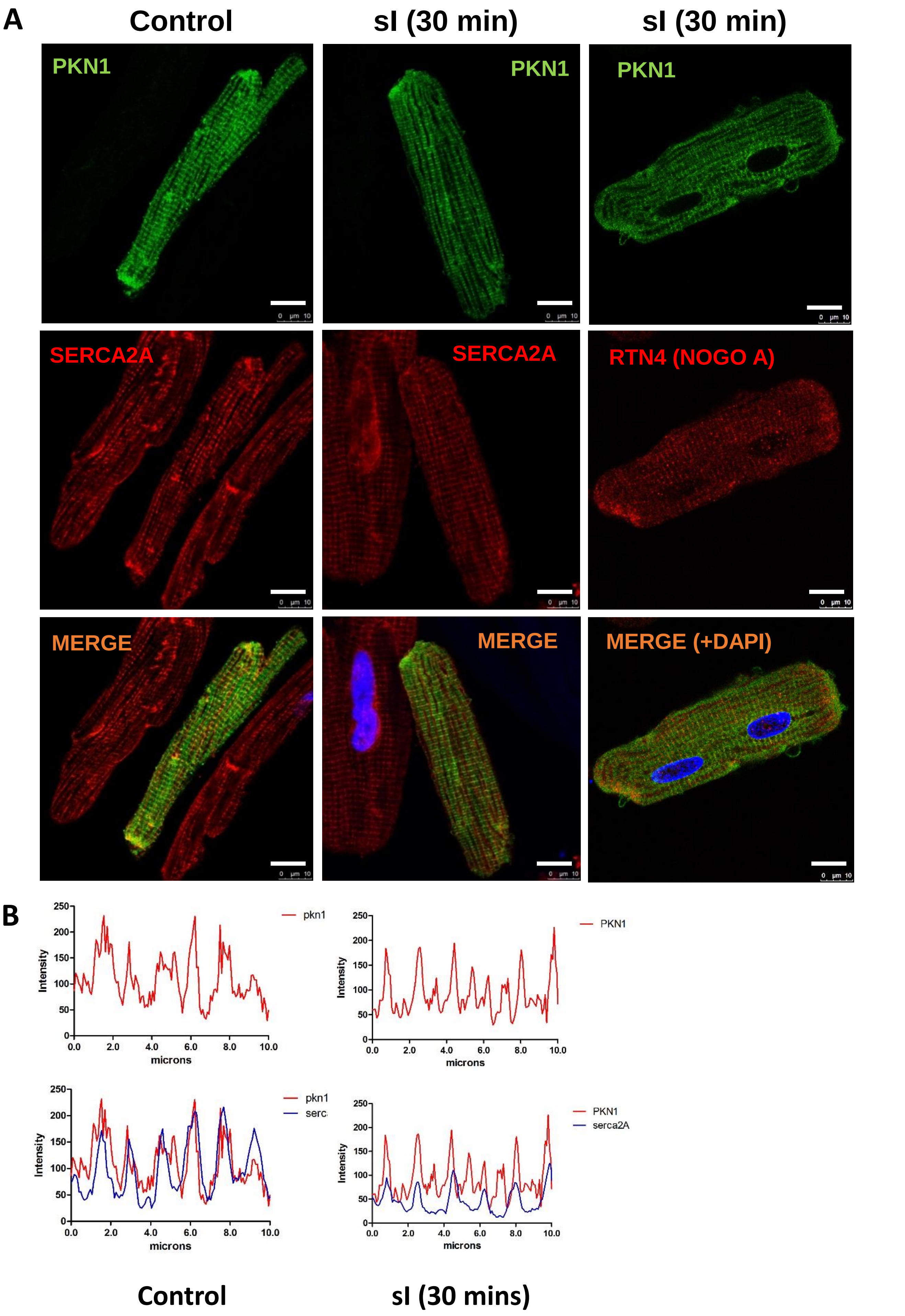
A
Control
sI (30 min)
sI (30 min)
PKN1
PKN1
PKN1
SERCA2A
SERCA2A
RTN4 (NOGO A)
MERGE
MERGE
MERGE (+DAPI)
B
pkn1
PKN1
Intensity
microns
pkn1
serc
PKN1
serca2A
Control
sI (30 mins)

Figure S5

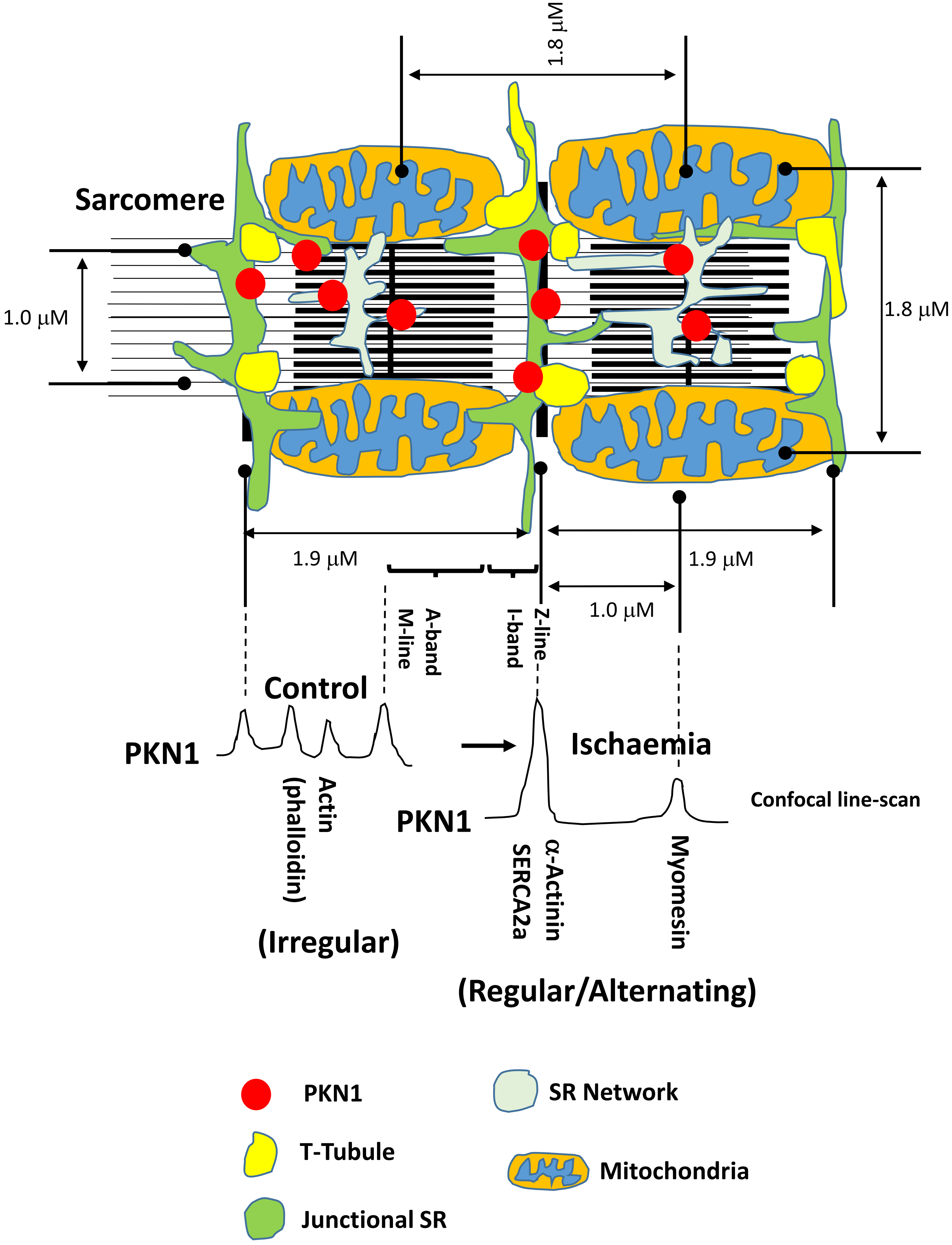

1.8 μM
Sarcomere
1.0 μM
1.8 μM
1.9 μM
1.9 μM
1.0 μM
M-line
A-band
I-band
Z-line
Control
PKN1
Actin (phalloidin)
(Irregular)
Ischaemia
PKN1
Confocal line-scan
α-Actinin
SERCA2a
Myomesin
(Regular/Alternating)
PKN1
T-Tubule
Junctional SR
SR Network
Mitochondria

Figure S6

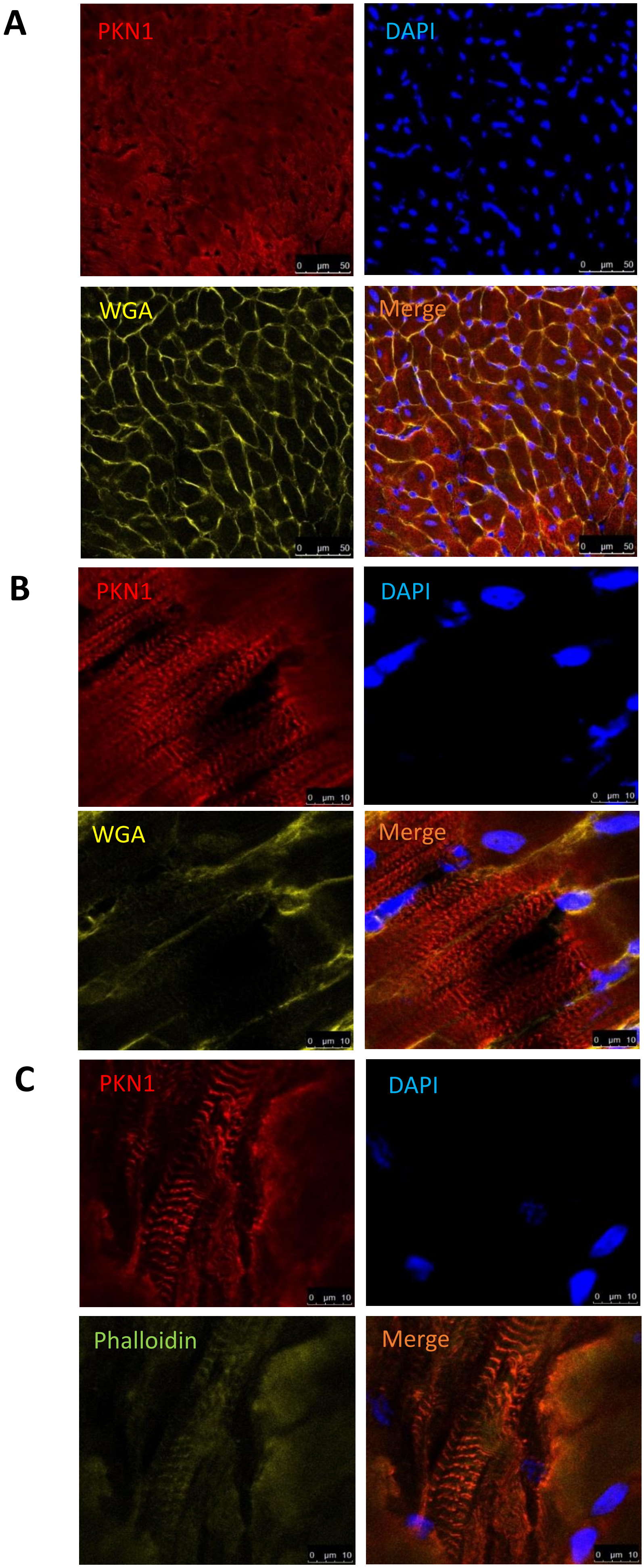
A
PKN1
DAPI
WGA
Merge
B
PKN1
DAPI
WGA
Merge
C
PKN1
DAPI
Phalloidin
Merge

Figure S7

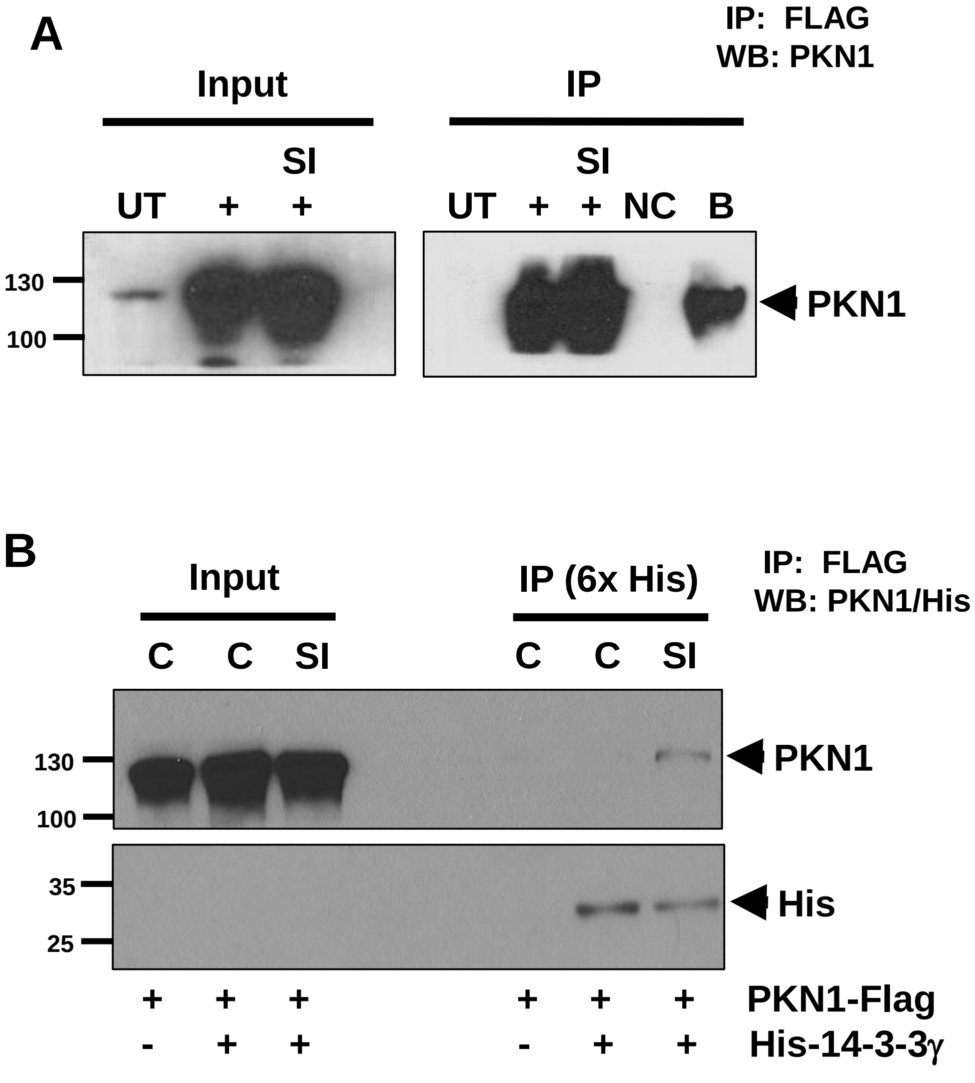
A
IP: FLAG
WB: PKN1
Input
IP
SI
SI
UT + + UT + + NC B
130
100
PKN1
B
Input
IP (6x His)
IP: FLAG
WB: PKN1/His
C C SI C C SI
130
100
PKN1
35
25
His
+ + + + + + PKN1-Flag
- + + - + + His-14-3-3γ

Figure S8

**In SI Only**<br><br>**Protein Threshold = 90%, Min Peptide = 2; Peptide Threshold = 90%**<br>**MS/MS View: Identified Proteins (18/173) Including 0 Decoys**	**Total Spectrum Count**	**% Coverage**
Adenyl cyclase-associated protein 1	18	39
Vigilin	9	8.1
Zyxin	8	11
Reticulon 4 (Nogo A)	8	12
Creatine kinase B type	6	17
Calreticulin	5	18
Ubiquitin carboxy-terminal hydrolase 5	4	8.3
E3 ubiquitin protein ligase (NEDD4)	6	12
Calcium-calmodulin dependent protein kinase II subunit delta (CAMK2D)	6	11
Reticulocalbin 2 (RCN2)	5	18
14-3-3 protein gamma (14-3-3γ)	3	19
Rho guanine nucleotide activator protein (RhoGAP)	2	7.1
Gelsolin	3	6.4
Alanine tRNA ligase cytoplasmic	3	4.6
PDZ and LIM domain protein 5	2	12
T complex protein 1 subunit theta (TCP1τ)	2	6.6
Reticulocalbin 1 (RCN1)	3	14
Creatine kinase type M	3	10

Supplementary Table ST1a

**<u>In controls Only</u>**<br><br>**Protein Threshold = 90%, Min Peptide = 2; Peptide Threshold = 90%**	**Total Spectrum Count**	**% Coverage**
Protein flightless-1 homolog	6	9.9
Tyrosine-protein kinase JAK1	3	3.3
Heterogenous nuclear ribonucleoprotein U	3	7.3
Unconventional myosin-XVIIIB	4	2.6
Leucine-rich repeat flightless interacting protein 1	3	7.0

Supplementary Table ST1b

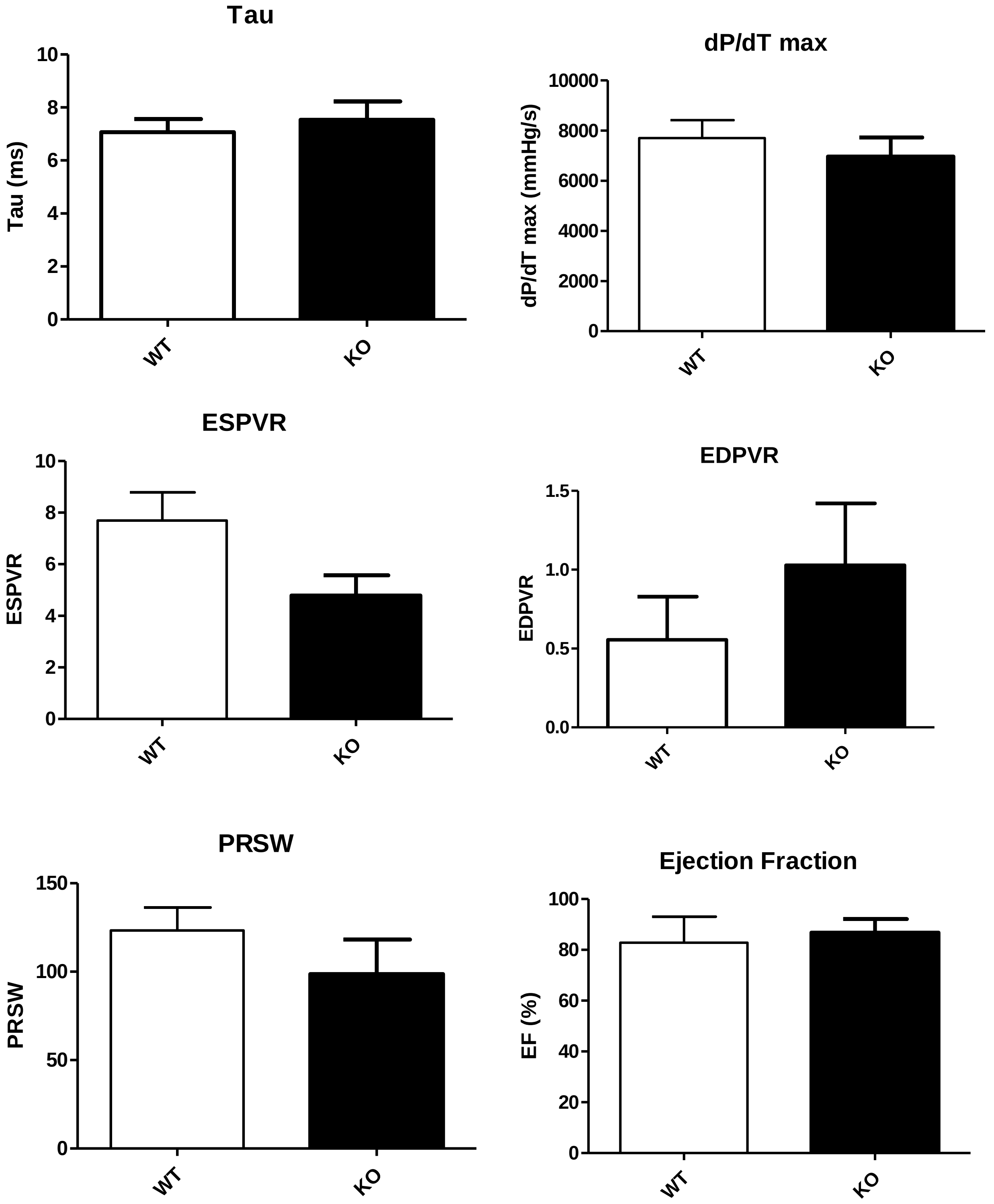
Tau
Tau (ms)
10
8
6
4
2
0
WT
KO
dP/dT max
dP/dT max (mmHg/s)
10000
8000
6000
4000
2000
0
WT
KO
ESPVR
ESPVR
10
8
6
4
2
0
WT
KO
EDPVR
EDPVR
1.5
1.0
0.5
0.0
WT
KO
PRSW
PRSW
150
100
50
0
WT
KO
Ejection Fraction
EF (%)
100
80
60
40
20
0
WT
KO

Figure S9

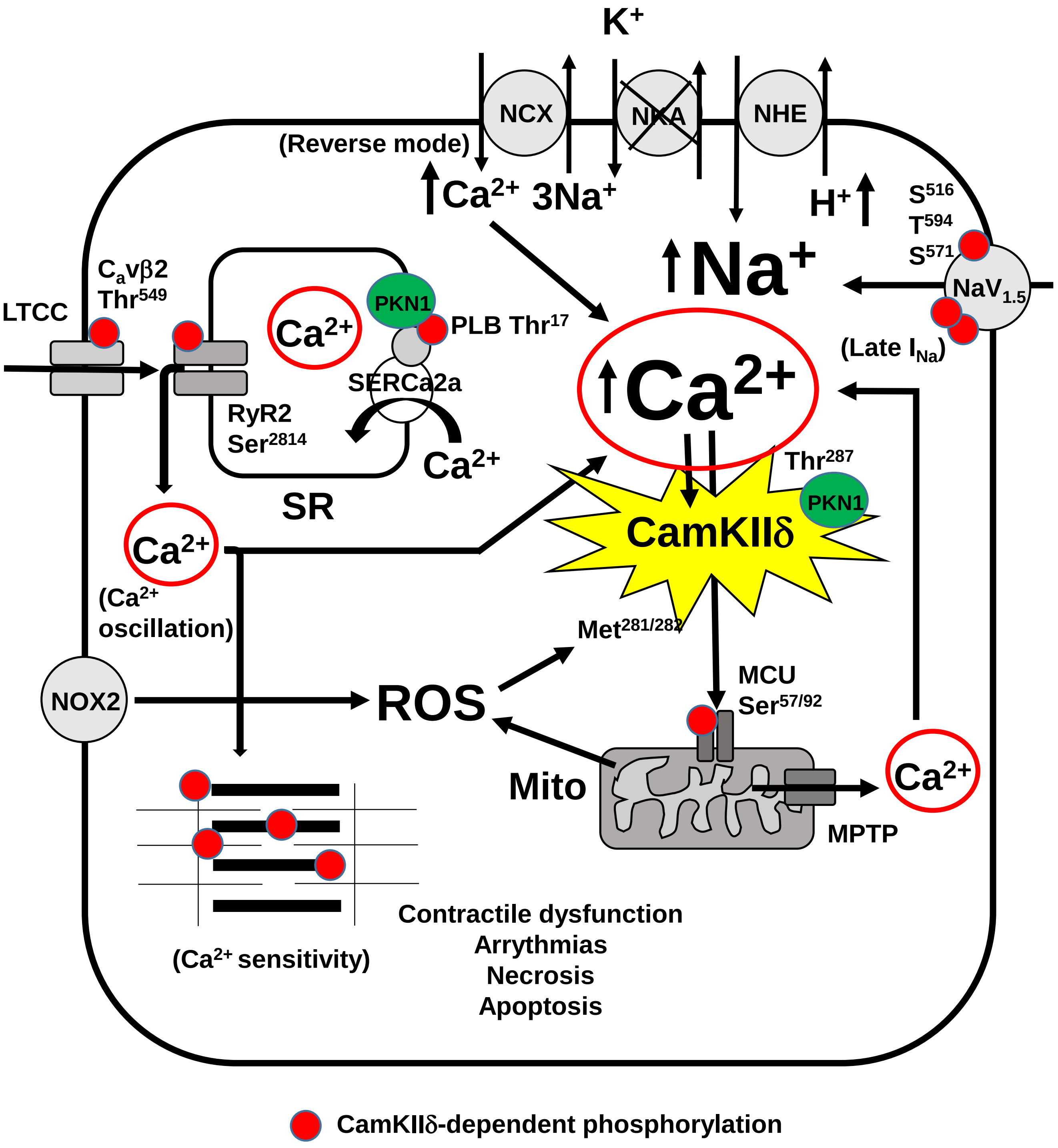
K+
NCX
NKA
NHE
(Reverse mode)
Ca2+ 3Na+
H+
S516
T594
S571
Na+
NaV1.5
(Late INa)
CavβAA2
Thr549
LTCC
PKN1
Ca2+
PLB Thr17
SERCa2a
RyR2
Ser2814
Ca2+
SR
Ca2+
Thr287
PKN1
CamKIIδ
Ca2+
(Ca2+
oscillation)
Met281/282
NOX2
ROS
MCU
Ser57/92
Mito
Ca2+
MPTP
(Ca2+ sensitivity)
Contractile dysfunction
Arrythmias
Necrosis
Apoptosis
CamKIIδ-dependent phosphorylation

Figure S9
