# Supplementary material for: Loss of Protein Kinase Novel 1 (PKN1) is associated with mild systolic and diastolic contractile dysfunction, increased phospholamban Thr17 phosphorylation, and exacerbated ischaemia-reperfusion injury
Source: Cardiovasc Res. 2017 Oct 16;114(1):138–57. doi: 10.1093/cvr/cvx206 (PMC5815577; doi:10.1093/cvr/cvx206)
Supplement: cvx206_Online_Supplementary_Information [file cvx206_online_supplementary_information.pdf]

Online Supplementary Information.

Supplementary Figure Legends.

Supplementary Figure S1: *Genotyping of PKN1 knockout mice and PKN1, PKN2 and PKN3 Expression.* Genotyping of PKN1 knockout mice by RT-PCR (left) and Western blots showing PKN1, PKN2 and PKN3 levels in wild type (+/+) and knockout (-/-) mice (right).

Supplementary Figure S2: *Baseline haemodynamic parameters in isolated WT and PKN1 KO hearts.* Hearts were perfused for 30 minutes of stabilisation (baseline comparison) prior to ischaemia and reperfusion. Values are presented as mean$\pm$s.e.m. (n=8) for left ventricular pressure (LVDP), end-diastolic pressure (EDP) and coronary flow (CF) recorded over 30 minutes. There were no significantly differences between genotypes of any baseline parameters.

Supplementary Figure S3: *Time Courses of MAPK/SAPK Activation during Ischemia-Reperfusion* in Mouse Hearts. **Panel A:** Schematic representation of ischemia-reperfusion time course protocols. Hearts were perfused in Langendorff mode and either harvested after 30 minutes stabilisation; following 30 minutes stabilisation hearts and 30 minutes global ischemia and harvested at various time points during ischemia or following 30 minutes stabilisation and 30 minutes global ischemia, hearts were reperfused and harvested at various time points during reperfusion. Arrows represent time points at which samples were harvested. **Panels B-D:** Time courses of MAPK/SAPK activation during ischemia-reperfusion in c57BL/6 male mice. Hearts were subjected to varying durations of ischemia (0-30minutes) and reperfusion (5-120minutes) before they were harvested for Western blot analysis. Activation of p42/p44 MAPK (**Panel B**), p46/p54 SAPK (**Panel C**) and p38 MAPK (**Panel D**) in response to ischemia-reperfusion were detected with dual phosphor-specific antibodies. Total p42/p44 MAPK, p46/p54 SAPK and p38 levels were assessed with corresponding pan-antibodies and used to normalise the phospho-MAPK/SAPK signal from the same sample for quantitation following densitometry. Data are mean±SEM derived from Western analysis of three independent experiments (three hearts per timepoint) where *$p<0.05$ v baseline (One way ANOVA).

Supplementary Figure S4: *MAPK/SAPK Activation in NRVMs during SI and reperfusion.* MAPKSAPK activation in NRVMs during SI was assessed at the indicated times of SI by Western immunoblotting using antibodies against dually phosphorylated p46/54-SAPK (JNK), p38-MAPK and p42/p44-MAPK (ERK1/2) (upper panels) and antibodies detecting total protein (lower panels) (**Panel A**). MAPKSAPK activation in NRVMs during SI and subsequent 'reperfusion' was assessed at the indicated times of 'reperfusion' by Western immunoblotting as for panel A. In each case NRVMs were treated with 50nM CLA as a positive control for phosphorylation (**Panel B**). Representative blots from three independent experiments are shown.

Supplementary Figure S5: *The co-localisation of PKN1 and Serca2a in ARVMs during SI.* **Panel A:** ARVMs were infected with adenovirus expressing WT-PKN1-FLAG and were either untreated (control) or subjected to 30 min SI prior to fixation and slide mounting. Slides were stained with mouse monoclonal anti-PKN1 antibody (green) and counterstained with antibodies against SR markers Serca2a antibody (red) or Reticulon 4 (Nogo A) (red) and DAPI (blue) to stain nuclei. Slides were analysed by confocal microscopy in the separate green, red and blue channels and a merged image is shown for the overlay of the individual images. In all cases experiments were repeated four times and >10 fields analysed per treatment. Scale bar equals 10μm. **Panel B:** Confocal line scans showing the intensity plots of the Cy3 (PKN1) and Cy5 (Serca2A) signals on the merged image. In all cases experiments were repeated four times and >10 fields analysed per treatment.

Supplementary Figure S6: *A schematic representation of the changes in PKN1 localisation.* Compared between control and simulated ischemia relative to the features of the myofilaments and SR visible on confocal images.

Supplementary Figure S7: *Confocal immunofluorescence images of endogenous PKN1 in sections of c57/Bl6 mouse heart LV*. Sections were stained with a mouse monoclonal antibody to PKN1 and Cy3™ goat-anti-mouse secondary (red) and co-stained with AlexaFluor™ 488-wheat germ haemagluttinin (WGA) (yellow); DAPI (blue) and AlexaFluor™ 488-phalloidin (green).

Supplementary Figure S8: Characterisation of *PKN1 Immunoprecipites and confirmation of binding to 14-3-3γ.* **Panel A:** NRVMs were either untransfected (UT) or transfected with WT-hPKN1-FLAG (+). Cells were then left untreated or treated with simulated ischaemia (SI) and immunoprecipitated (IP) with an anti-FLAG antibody. IPs were Western blotted and probed for PKN1 using an anti-PKN1 antibody. **Panel B**: NRVM were transfected with WT-hPKN1-FLAG and then cell lysates prepared. Cell lysates were then spiked with purified recombinant 6xHis-tagged 14-3-3γ and IP using anti-FLAG. IPs were Western blotted and probed for PKN1 (anti-PKN1) or His-14-3-3γ (anti-His). Representative blots from three independent experiments.

Supplementary Table 1: *Analysis of Binding Partners of PKN1during SI in NRVMs.* NRVMs were transduced with adenovirus expressing PKN1-FLAG and treated with SI for 20 minutes. Samples were harvested and PKN-FLAG immunoprecipitated using anti-FLAG antibody. Candidate PKN1 binding proteins in the immunoprecipitates were identified by mass spectrophotometric analysis (LC-MS/MS) as described in methods. Data was analysed under high stringency conditions where only proteins identified as unique to either control only or ischemia only are shown and parameters were set to include only >95% matches for a minimum of three corresponding peptides in three separate samples (n=3).

Supplementary Figure S9: *Analysis of baseline cardiac performance in wild type versus knockout hearts.* Cardiac functional parameters: speed of relaxation (Tau);

maximal rate of pressure development (dP/dT max); End Systolic Pressure Volume Relationship (ESPVR); End Diastolic Pressure Volume Relationship (EDPVR); Preload-recruitable stroke work (PRSW) and Ejection Fraction were determined by PV loop analysis using an admittance catheter (Scisense®) inserted into the left ventricle under anaesthesia. Statistical analysis was by One Way ANOVA where significance was taken as *$p \leq 0.05$ versus WT where n=5.

Supplementary Figure S10: *Sites of PKN1 Action During Ischemia/Reperfusion*. A schematic representation of the key events leading to Ca^{2+} overload in cardiomyocytes and where PKN1 may interfere with CamKIIδ-dependent PLB Thr^{17} phosphorylation.
